# Hinged Carboxylate in the Artificial Distal Pocket of an Iron Porphyrin Enhances CO_2_ Electroreduction at Low Overpotential

**DOI:** 10.1002/advs.202500482

**Published:** 2025-01-22

**Authors:** Adrien Smith, Philipp Gotico, Régis Guillot, Stéphane Le Gac, Winfried Leibl, Ally Aukauloo, Bernard Boitrel, Marie Sircoglou, Zakaria Halime

**Affiliations:** ^1^ Université Paris‐Saclay CNRS Institut de Chimie Moléculaire et des Matériaux d'Orsay Orsay 91400 France; ^2^ Institute for Integrative Biology of the Cell CEA CNRS Université Paris‐Saclay Gif‐sur‐Yvette 91191 France; ^3^ Univ Rennes CNRS ISCR (Institut des Sciences Chimiques de Rennes)‐UMR 6226 Rennes 35000 France

**Keywords:** artificial distal pocket, biomimetic, carbon dioxide, electrocatalysis, hydrogen bonding

## Abstract

To efficiently capture, activate, and transform small molecules, metalloenzymes have evolved to integrate a well‐organized pocket around the active metal center. Within this cavity, second coordination sphere functionalities are precisely positioned to optimize the rate, selectivity, and energy cost of catalytic reactions. Inspired by this strategy, an artificial distal pocket defined by a preorganized 3D strap is introduced on an iron‐porphyrin catalyst (**sc‐Fe**) for the CO_2_‐to‐CO electrocatalytic reduction. Combined electrochemical, kinetic, and computational studies demonstrate that the adequate positioning of a carboxylate/carboxylic group acting in synergy with a trapped water molecule within this distal pocket remarkably enhances the reaction turnover frequency (TOF) by four orders of magnitude compared to the perfluorinated iron‐tetraphenylporphyrin catalyst (**F_20_Fe**) operating at a similar low overpotential. A proton‐coupled electron transfer (PCET) is found to be the key process responsible for the unexpected protonation of the coordinating carboxylate, which, upon CO_2_ insertion, shifts from the first to the second coordination sphere to play a possible secondary role as a proton relay.

## Graphical abstract

Enzyme‐like behavior of an artificial distal pocket formed by a preorganized 3D strap on an iron‐porphyrin enhances the reaction rate of CO_2_‐to‐CO electrocatalytic reduction at low overpotential. Electrochemical, kinetic, and computational studies demonstrate that a strategically positioned hanging carboxylic group along with a trapped water molecule within this distal pocket facilitate CO_2_ insertion in the rate‐determining step of the reaction mechanism.

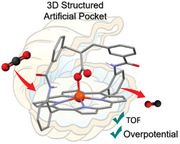

## 1 Introduction

**Figure 1:**
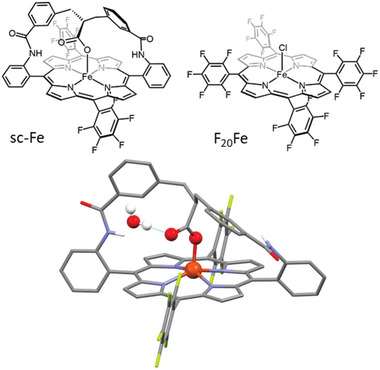
Molecular structures of the catalysts **sc‐Fe** and **F_20_Fe** (top) and X‐ray crystal structure of catalyst **sc‐Fe** (bottom, CCDC 2363623).

One of the pivotal challenges in energy research revolves around finding practical and cost‐efficient means to store electrical energy in chemical bonds, with CO_2_ serving as a primary starting material.^[^ ^1^, ^2^, ^3^, ^4^, ^5^, ^6^ ^]^ Within this context, the focus has increasingly turned toward homogeneous catalysts for the electrocatalytic CO_2_ reduction reaction (CO_2_RR).^[^ ^7^, ^8^, ^9^, ^10^, ^11^, ^12^, ^13^, ^14^ ^]^ These highly structured catalysts present the opportunity to delve deep into the reaction mechanisms and enable chemists to fine‐tune catalytic performances through iterative ligand modifications. If the primary coordination sphere rules the redox properties of these catalysts’ metal center toward CO_2_ reduction, more subtle effects have recently been discovered through modifications of the secondary coordination sphere (SCS), *i.e*., chemical functions that are not directly linked to the metal center, resulting in enhanced rate and selectivity at lower overpotential.^[^ ^15^, ^16^, ^17^ ^]^ These functions can play various roles such as proton relay (phenols,^[^ ^18^ ^]^ amines,^[^ ^19^ ^]^ carboxylic acids^[^ ^20^, ^21^ ^]^), H‐bond donors (amines,^[^ ^22^ ^]^ amides,^[^ ^23^ ^]^ guanidine,^[^ ^24^ ^]^ ureas^[^ ^25^, ^26^, ^27^ ^]^), electrostatic intermediate stabilizers (ammonium cations,^[^ ^28^ ^]^ and imidazolium moieties^[^ ^29^, ^30^ ^]^), or Lewis acids (bimetallic complexes^[^ ^31^, ^32^ ^]^). The implementation of such functionalities was mainly inspired by the surroundings of enzymatic catalytic sites for their exceptional performances. The structure of the distal pocket hosting the SCS within the enzyme's active sites plays an important role in the activation and transformation of small molecules. For example, extensive research has focused on understanding the significance of the well‐defined structure and precisely positioned amino acid residues and metal centers within this pocket, particularly in the context of dioxygen (O_2_) activation in enzymes such as P450 and Cytochrome *c* Oxidase.^[^ ^33^, ^34^, ^35^, ^36^ ^]^ By recognizing the critical role of such preorganized architecture in creating a specific and well‐defined local environment and minimizing reorganizational energy during various reaction steps such as substrate capture, activation, proton transfer and product release, chemists have developed synthetic models for O_2_ activation and reduction incorporating a more and more rigid and preorganized SCS.^[^ ^37^ ^]^ Iron‐porphyrins stand out among these models, offering a relatively rigid platform where functional groups on the *ortho* position of the porphyrin *meso* phenyl groups can be strategically employed to construct an artificial distal pocket above the iron center.^[^ ^38^, ^39^, ^40^, ^41^, ^42^ ^]^ Successful applications of this approach have been demonstrated in tuning the rate and selectivity of catalytic reactions involving O_2_ activation. However, this strategy has yet to be explored in the context of catalytic CO_2_ reduction by iron‐porphyrins. Nocera et al. have recently reported on the deleterious effect that a carboxylate group in the SCS of a hangman iron‐porphyrin catalyst has on the rate of electrocatalytic CO_2_RR.^[^ ^21^ ^]^ This effect was found to arise from repulsive electrostatic interactions between the negatively charged carboxylate and the Fe‐CO_2_ adduct.^[^ ^43^ ^]^ In this work, we demonstrate that when such a “hanging” carboxylate group is integrated into a meticulously constructed artificial distal pocket, formed using a 3D preorganized strap (**sc‐Fe**, where **sc** stands for strapped carboxylate, **Figure** **1**), it remarkably enhances the reaction turnover frequency (TOF) by four orders of magnitude compared to the perfluorinated iron‐porphyrin catalyst (**F_20_Fe**) operating at a similar low overpotential. Kinetic and computationnal mechanistic studies reveal that the coordinating carboxylate group in **sc‐Fe** undergoes protonation via a proton‐coupled electron transfer (PCET) process promoting the insertion of CO_2_ in the rate determining step of the reaction mechanism, with the assistance of a trapped water molecule within the distal pocket.

## 2 Results and Discussion

Catalyst **sc‐Fe** was synthesized by adapting our previously reported synthetic strategy for a non‐fluorinated analog.^[^ ^44^ ^]^ Perfluorinated aryl groups were incorporated here on the two unstrapped *meso* positions of the macrocycle to shift the redox potentials of the catalyst to more positive values matching the electrochemical window of the **F_20_Fe** reference catalyst. All synthetic procedures (Figure S1) and characterizations (Figures S34–S61) are gathered in the Supporting Information. X‐ray diffraction analysis of **sc‐Fe** was performed on single crystals obtained by slow evaporation of a saturated solution of the complex in an acetone/methanol/water solvent mixture (Figures 1 and S2, and Table S1, Supporting Information for a more detailed description of the structure). **sc‐Fe** structure shows the Fe(III) cation penta‐coordinated by the four N‐atoms of the porphyrin with an average Fe‒N distance of 2.06 Å and one O‐atom of the carboxylated group in an *η* ^1^(O) carboxylato binding mode with a Fe‒O distance of 1.88 Å. In this square pyramidal first coordination sphere, as expected for a high spin iron‐porphyrin, the Fe center is positioned 0.45 Å above the plane defined by the 24 atoms of the porphyrin rim. More importantly, only a limited average deviation of 0.04 Å from this plane can be measured, highlighting the well‐thought‐out design of the pre‐organized strap, imposing only minimal constraints and distortion on the porphyrin platform. The structure also reveals that the distal pocket, created by the strap above the metal center, hosts a trapped water molecule maintained in position by H‐bonding interaction with the non‐coordinating O‐atom of the overhanging carboxylate group.

**Figure 2:**
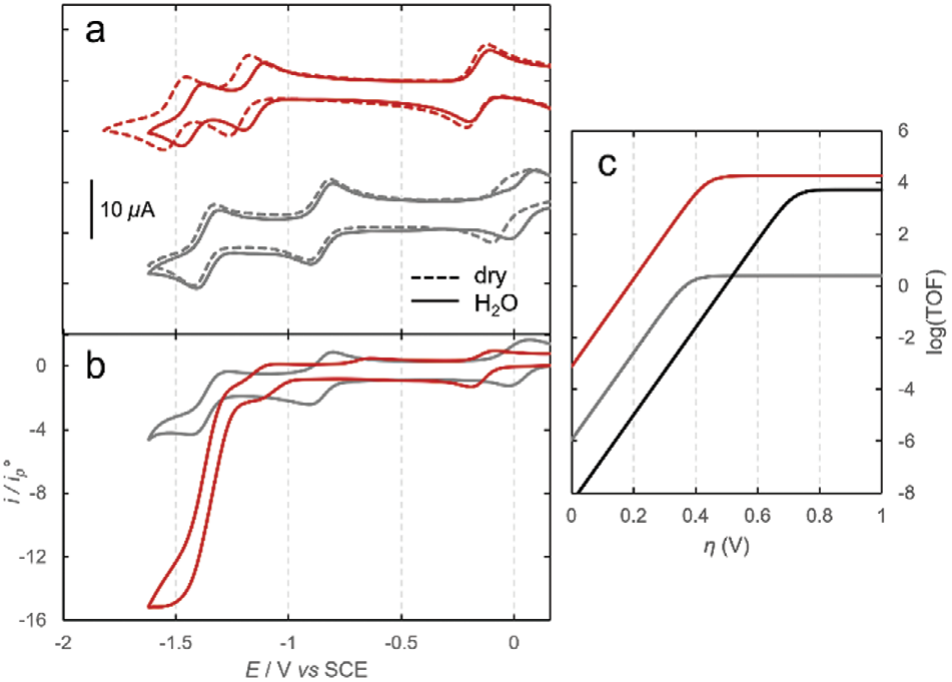
a) Cyclic voltammograms of modified iron porphyrin complexes under investigation: **sc‐Fe** (red), **F_20_Fe** (gray), under argon at a concentration of 1 mm in dry dimethylformamide (DMF) (dashed curve) or in the presence of 5 m of water (continuous curve) containing 0.1 m [Bu_4_N]PF_6_ at 25 °C, b) under CO_2_ with 5 m of water as proton source. c) Tafel plots of **sc‐Fe** (red), **F_20_Fe** (gray), and **TPP‐Fe** (black) calculated from FOW analysis. All color legends for (b) and (c) correspond to the same indications as in (a). Further details of FOW analysis are reported in the Supporting Information.

The redox properties and catalytic activity of **sc‐Fe** were then investigated by combining electrochemical, kinetic, and computational studies. Cyclic Voltammogram (CV) of **sc‐Fe** (1 mM) in argon‐degassed dry dimethylformamide (DMF) containing 0.1 m of tetra‐N‐butylammonium hexafluorophosphate ([Bu_4_N]PF_6_) shows three reversible redox waves corresponding to the formal Fe(III/II), Fe(II/I), and Fe(I/0) couples (**Figures** **2**a, S3 and Table S2, Supporting Information). As anticipated, the cumulative electron withdrawing effect of the two perfluorinated aryl groups and the two amide groups holding the strap in **sc‐Fe** add‐up to an inductive effect similar to that of the four perfluorinated aryl groups in **F_20_Fe**, resulting in comparable redox potentials window (Figure 2a, Table S2, Supporting Information).

**Figure 3:**
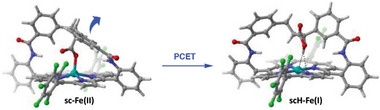
PCET event at **sc‐Fe(II)** triggers the unfolding of the strapped carboxylic moiety with partial release of the coordination site at Fe. The dots indicate non‐covalent interactions (see Figures S27–S29, Supporting Information).

Upon the addition of 5 m of water, the CVs of the two catalysts display two notable differences. The first is related to the Fe(III/II) redox wave that remains unchanged in the case of **sc‐Fe** (E = −0.14 V vs saturated calomel electrode (SCE)) but shifts anodically by 50 mV in **F_20_Fe** (E = ‒0.02 to +0.03 V) (Figure 2a, Figures S3 and S6, Table S2, Supporting Information). This shift is the result of a Cl‾ axial ligand exchange with a OH‾ coming from water.^[^ ^45^ ^]^ The absence of a shift in the case of **sc‐Fe** suggests that the intramolecular carboxylate ligand is not displaced by OH‾ and remains coordinated to the metal center. Accordingly, the geometries of **sc‐Fe(III)** and **sc‐Fe(II)** optimized using density functional theory (DFT) reveal that the distance between the metal and the *η* ^1^‐bound carboxylate only elongates by 5% upon this first reduction event (Figure S25, Supporting Information). The second difference between the CVs of the two catalysts concerns the important anodic shifts in the potentials corresponding to Fe(II/I) and Fe(I/0) redox couples of **sc‐Fe** by 40 and 60 mV respectively, whereas no significant change is observed for **F_20_Fe**. An even more pronounced shift of 120 mV is observed at the Fe(II/I) redox wave when water is replaced by trifluoroacetic acid (TFE) as a more acidic proton source (Figure S8, Table S3, Supporting Information). A plausible explanation for these shifts can be the protonation of the bonded carboxylate group, decreasing the electronic charge of the complex, thus facilitating its reduction at more positive potentials. It is important to mention however that given its low p*K*_a_ (≈14 in DMF) the carboxylate is unlikely to undergo a simple protonation by water (p*K*_a_ = 31.5) or TFE (p*K*_a_ = 24.0).^[^ ^46^ ^]^ These results therefore suggest a PCET linking the reduction of the Fe(II) to the formal Fe(I) state, to the protonation of the carboxylate group. Our computational study supports this hypothesis (Figure S26, Supporting Information). Indeed, without protonation, a potential of ‒1.22 V versus SCE is predicted to further reduce the axially bound **sc‐Fe(II)** species. In comparison, the potential calculated for the **sc‐Fe(II)** to **scH‐Fe(I)** PCET using TFE as a proton source is positively shifted to ‒1.03 V versus SCE, in good agreement with our experimental observations. Interestingly, this PCET also lead to an increase in the distance between the Fe center and the carboxylic O atom from 2.00 to 2.37 Å which is sufficient to render the unfolding of the diphenylpropyl strap accessible together with a steric decompression around the metal center in **scH‐Fe(I)** (**Figures** **3** and S27, Supporting Information).

Under a CO_2_ atmosphere, both **sc‐Fe** and **F_20_Fe** catalysts exhibit a catalytic response near the third redox wave corresponding to the two‐electron reduction of CO_2_ to CO. However, the intensity of the catalytic current in the case of **sc‐Fe** is more than five times higher than that of **F_20_Fe** (Figure 2b, Figures S5 and S7, Supporting Information). The production of CO was confirmed in bulk electrolysis experiment where gas products were quantified using gas chromatography (see Supporting Information with summary of Faradaic efficiency on Table S8). Figure 2c compares the Tafel plots [log(TOF) vs overpotential (*η*)] determined using Foot of the Wave (FOW) analysis on these two catalytic waves and that of the parent nonfunctionalized iron‐tetraphenylporphyrin catalyst (**TPP‐Fe)** reported in previous studies.^[^ ^29^ ^]^ As expected, electron withdrawing groups on **sc‐Fe** and **F_20_Fe** lead to a gain of more than 330 mV in the reaction overpotential compared to **TPP‐Fe**. In **F_20_Fe**, the counterpart of this through‐bond inductive effect on the first coordination sphere is a four orders of magnitude slower reaction rate (log(TOF_max_) = 0.39) than that of **TPP‐Fe** (log(TOF_max_) = 3.73). To our satisfaction, **sc‐Fe** exhibits a remarkably high log(TOF_max_) value of 4.26 pointing toward a second coordination sphere effect induced by the overhanging carboxylate/carboxylic group. These findings stand in sharp contrast to a recent study where a significant decrease in the reaction rate was observed for a hangman iron‐porphyrin featuring a carboxylic group in the SCS.^[^ ^21^ ^]^ In this study, the non‐coordinating carboxylate group – formed after the first TON – was suggested to disfavor CO_2_ binding in the rate‐determining step of the reaction mechanism due to through space electrostatic repulsion between the negatively charged carboxylate and the captured CO_2_.^[^ ^43^ ^]^ Here, our computations show that the carboxylic group interacts through hydrogen bonding with the CO_2_ adduct already in the Fe(I) state.^[^ ^47^ ^]^ This finding aligns with our previous report on a similar early activation of CO_2_ observed in an iron‐porphyrin catalyst featuring hydrogen bonding urea groups in the SCS.^[^ ^48^ ^]^

**Figure 4:**
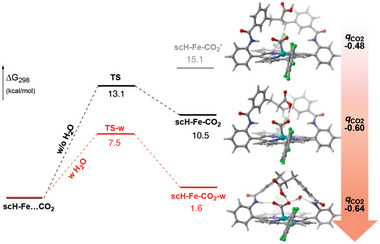
Left: Energetic profile for the binding of CO_2_ in the presence (red) or absence (black) of a trapped water molecule. Middle: Computed structures of the **scH‐Fe‐CO_2_** adducts; the dots indicate H‐bonds. Right: Comparison of the NPA charges at the CO_2_ fragment depending on the presence of H‐bonding with the strapped carboxylic moiety and the water molecule. In **scH‐Fe‐CO_2_′** the proton of the carboxylic acid was directed away from the CO_2_ moiety to prevent the H‐bonding.

To shed some light on the reaction mechanism, deuterium labelling experiments were performed. A significant Kinetic Isotope Effect (KIE = 4.2; H_2_O/D_2_O) was observed (Figure S17, Table S7, Supporting Information). On the other hand, when TFE or phenol (p*K*_a_ = 18.8) were used as stronger proton sources, slower reaction rates were measured (Figure S16, Supporting Information). Taken together, these results may point to a water‐involved rather than a proton transfer rate‐determining step. Inspired by the presence of a hydrogen‐bonded water molecule in the crystal structure of **sc‐Fe** (Figure 1), we investigated the possible role of water in the CO_2_ trapping/activation process using DFT. We first studied the coordination of CO_2_ by the **scH‐Fe(I)** intermediate, in the absence of water. This step is found to be endergonic by 10.5 kcal mol^−1^, with an activation barrier of 13.1 kcal mol^−1^ (**Figure** **4**). Alternatively, the inclusion of a water molecule within the distal pocket not only stabilizes the **scH‐Fe(I)‐CO_2_** by 9 kcal mol^−1^, but also decreases the activation barrier by almost half. This stabilization of the **scH‐Fe(I)‐CO_2_** adduct can be attributed to the establishment of two additional hydrogen bond interactions with the CO_2_ ligand, involving an amido group on one side, and a water molecule bound to the second amido group on the other side (Figure S32, Supporting Information). The role of water is therefore crucial here in adjusting the size of the distal pocket to host the CO_2_ molecule and facilitate its activation. In addition, water helps pre‐organizing the catalytic pocket to proceed through a more reactant‐like transition state, which is lower than that computed in absence of water.

Notably, the hydrogen‐bond interaction induced by the carboxylic strap within the distal pocket of **scH‐Fe‐CO_2_** was found to exert an important pull effect on the oxygen atoms of the bound CO_2_ leading to an increase of its negative charge by 25%. This negative charge shift even reaches a total of 33% when considering also the effect of the water molecule (Figure S31, Supporting Information). A desymmetrization of the CO_2_ molecule is also observed, which should facilitate the subsequent reduction and protonation steps. These effects were further investigated by DFT calculaionin order to identify the possible reaction sequence and explore a prospective role of the carboxylic group as a proton relay. The results are presented in Figure S33 (Supporting Information). First, it is interesting to note that no protonation occurs from the carboxylic acid group to the bound CO_2_, even upon reduction. This precludes the formation of a pending carboxylate group under our catalytic conditions, in contrast with the previous study of Nocera et al.^[^ ^43^ ^]^ We therefore considered that the proton should come from the most acidic proton source available in solution, i.e., H_2_CO_3_, and could proceed either in a concerted way through the hanging carboxylic group, or via the direct protonation of CO_2_. An alternative pathway, which was not considered in this study, would be the protonation through a water bonded molecule. Our results show that the protonation of the CO_2_ fragment cannot proceed without a prior or a concomitant extra 1e^−^ reduction. Accordingly, from **scH‐Fe(III)‐CO_2_**, three 1e^−^ processes are thermodynamically accessible at the catalytic potential range. One is the sequential electron transfer and proton transfer (ETPT) involving reduction of **scH‐Fe(III)‐CO_2_** (−1.07 V versus SCE) followed by its direct protonation (−5.6 kcal mol^−1^), the two others less favorable pathways are concerted PCET and are predicted at −0.95 and −0.75 V versus SCE. The second protonation is then driven by the release of a water molecule, via a direct or a domino proton transfer (Figure S33, Supporting Information). Further study would be needed to evaluate the activation energy of these steps, but given their exergonicity, very low barriers are expected, rendering the two pathways competitive.

**Figure 5:**
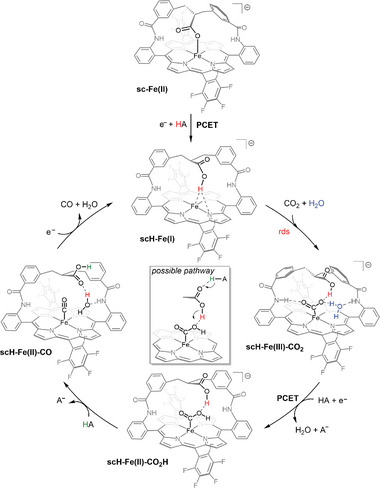
Proposed mechanism for the CO_2_ to CO electrocatalytic reduction by **sc‐Fe** catalyst.

Putting all the gathered computational, electrochemical, and kinetic data together, we can propose a plausible mechanism to rationalize the superior catalytic performance of **sc‐Fe** for CO_2_‐to‐CO electrocatalytic reduction (**Figure** **5**). The mechanism begins with the 1e^−^ reduced form of the catalyst **sc‐Fe(II)**, where the iron center is axially coordinated by the hanging carboxylate. A PCET process then leads to the formation of **scH‐Fe(I)** as the catalytic active species. This process induces a conformation change, causing the strap to unfold and the Fe···O distance to elongate, thereby opening the distal pocket to host the CO_2_ substrate (Figure 3). To some extent, this behavior is similar to that observed in the active site of molybdenum/tungsten‐containing formate dehydrogenase, where a metal‐coordinated selenocysteine residue leaves the first coordination sphere upon reduction and protonation, making room for the insertion of CO_2_.^[^ ^2^, ^49^ ^]^ In our case, this insertion of CO_2_ to form **scH‐Fe(III)‐CO_2_** intermediate, identified as the rate‐determining step of the mechanism, is also facilitated by both the hanging carboxylic group and a trapped water molecule within the distal pocket (Figure 4). Subsequently, a second favorable PCET process is computed to lead to the formation of the **scH‐Fe(II)‐CO_2_H** species. Our computations also indicate that the carboxylic group in the SCS may play another role in the following protonation step, acting as a proton relay between the exogenous acid source (H_2_CO_3_) and the catalytic center. This last protonation triggers C─O bond cleavage and the formation of **scH‐Fe(II)‐CO** intermediate. Finally, in the last step, CO and a water molecule are released upon 1e^−^ reduction to regenerate the **scH‐Fe(I)** active species.

## 3 Conclusion

In conclusion, our combined experimental and computational study demonstrates that when strategically positioned within a well‐defined artificial distal pocket of an iron‐porphyrin catalyst (**sc‐Fe**), a carboxylate moiety can significantly enhance the CO_2_‐to‐CO electrocatalytic reduction, increasing the TOF by four orders of magnitude compared to the perfluorinated iron‐tetraphenylporphyrin catalyst (**F_20_Fe**) operating at a similar low overpotential. The structural arrangement imposed by this distal pocket favors the protonation of the coordinating carboxylate via a PCET process, yielding the **scH‐Fe(I)** catalytic active species. In an enzyme‐like behavior, this process is accompanied by a conformational change that facilitates CO_2_ substrate insertion. In this CO_2_ capture process, the carboxylic moiety shifts from the first to the second coordination sphere and, together with a water molecule trapped by the distal pocket, plays a crucial role in assisting CO_2_ binding and activation at the Fe(I) oxidation state. A possible secondary role as proton relay was also attributed to this hanging carboxylic group. This first exploration of the concept of confinement within a weakly binding artificial cavity for CO_2_ electroreduction by iron‐porphyrins sets the stage for future spectroscopic and theoretical studies to examine further the role of this proximal pocket in substrate orientation and activation, proton transfer, C─O bond cleavage and product release. The goal is to extract the key features offered by metalloprotein binding cavities to overcome the main bottlenecks of current synthetic models.

## Conflict of Interest

The authors declare no conflict of interest.

## Supporting information



Supporting Information

## Data Availability

The data that support the findings of this study are openly available in [Cambridge Crystallographic Data Centre] at [http://www.ccdc.cam.ac.uk/structures/], reference number [2363623].
