# Supplementary material for: Hinged Carboxylate in the Artificial Distal Pocket of an Iron Porphyrin Enhances CO2 Electroreduction at Low Overpotential
Source: Adv Sci (Weinh). 2025 Jan 22;12(11):2500482. doi: 10.1002/advs.202500482 (PMC11923859; doi:10.1002/advs.202500482)
Supplement: Supplementary file 1 — Supporting Information [file ADVS-12-2500482-s001.docx]

Supporting Information

Hinged Carboxylate in the Artificial Distal Pocket of an Iron Porphyrin Enhances CO_2_ Electroreduction at Low Overpotential

Adrien Smith,^[a]^ Philipp Gotico,^[b]^ Régis Guillot,^[a]^ Stéphane Le Gac,^[c]^ Winfried Leibl,^[b]^ Ally Aukauloo,^[a,b]^ Bernard Boitrel,^[c]^* Marie Sircoglou,^[a]^* and Zakaria Halime^[a]^*

[a] A. Smith, Dr. R. Guillot, Prof. A. Aukauloo, Dr. Marie Sircoglou, Dr. Z. Halime

Université Paris-Saclay, CNRS, Institut de Chimie Moléculaire et des Matériaux d'Orsay, 91400, Orsay, France.

E-mail: marie.sircoglou@universite-paris-saclay.fr, zakaria.halime@universite-paris-saclay.fr

[b] Dr. P. Gotico, Prof. A. Aukauloo, Dr. W. Leibl,

Institute for Integrative Biology of the Cell, CEA, CNRS, Université Paris-Saclay, 91191, Gif-sur-Yvette, France.

[c] Dr. S. Le Gac, Dr. B. Boitrel,

Univ Rennes, CNRS, ISCR (Institut des Sciences Chimiques de Rennes), UMR 6226, 35000, Rennes, France

E-mail: bernard.boitrel@univ-rennes.fr

**Table of Contents**

[Abbreviations 3](#_Toc182497218)

[I. Synthesis and characterization 4](#_Toc182497219)

[Materials and methods 4](#_Toc182497220)

[Synthesis of porphyrin ligands and iron porphyrins 4](#_Toc182497221)

[II. X-Ray Diffraction Crystallography 8](#_Toc182497222)

[III. Electrochemical analysis 10](#_Toc182497223)

[Cyclic Voltammetry 10](#_Toc182497224)

[General methods and materials 10](#_Toc182497225)

[Determination of *k*_cat_ (Foot-of-the-Wave Analysis) 16](#_Toc182497226)

[Kinetic Isotopic Effect (KIE) 22](#_Toc182497227)

[Catalyst Diffusion Coefficient 24](#_Toc182497228)

[Controlled potential electrolysis (CPE) 25](#_Toc182497229)

[Materials and setup 25](#_Toc182497230)

[Faradic Efficiency, selectivity and TON 26](#_Toc182497231)

[IV. UV-visible of sc-Fe 29](#_Toc182497232)

[V. Computational Details 30](#_Toc182497233)

[VI. Spectroscopic data (NMR and HRMS spectra) 37](#_Toc182497234)

[References 65](#_Toc182497235)

# Abbreviations

**ACN** Acetonitrile

**CPE** Controlled Potential Electrolysis

**CV** Cyclic Voltammetry

**DCM** Dichloromethane

**DMF** N,N-dimethylformamide

**F_20_FeTPP** 5,10,15,20-tetra-(pentafluorophenyl)porphyrinato iron(III) chloride

**Fc / Fc^+^** Ferrocene / Ferrocenium

**SCE** Standard Calomel Electrode

**TBAPF_6_** Tetrabutylammonium hexafluorophosphate (Bu_4_NPF_6_)

**TFE** 2,2,2-trifluoroethanol

**THF** Tetrahydrofuran

**TOF** Turnover frequency

**TON** Turnover number

# Synthesis and characterization

## Materials and methods

Mass spectra (ESI: *Micromass MS/ MS ZABSpec TOFF* spectrometer MALDI-TOF: *Microflex-LT Bruker Daltonics*) were performed at the C.R.M.P.O. (University of Rennes 1). ^1^H-, ^19^F- and ^13^C-NMR spectra were recorded either on *BrukerAvance500* or *BrukerAvance400* spectrometers equipped with a BBFO probe. Spectra were referenced with residual solvent protons. UV-Vis spectra were recorded on an Uvikon XL spectrometer. Chemicals were purchased from Aldrich and were used as received. Solvents were purchased form Aldrich or VWR and were used as received. THF (K/benzophenone, N_2_) was distilled before use.

## Synthesis of porphyrin ligands and iron porphyrins

Figure S1. Synthesis pathway for strapped carboxylate iron porphyrin sc-Fe (see below for detail).

**5-(pentafluorophenyl)-dipyrromethane** **6** was prepared according to literature.^[[1]](#endnote-1)^

1. **5,15-bis-(2-nitrophenyl)-10,20-bis-(pentafluorophenyl)-porphyrin 5**. 5-(pentafluorophenyl)-dipyrromethane **6** (25.3 mmol, 7.9 g) and 2-nitrobenzaldhyde (3.85 g, 1 equiv.) were dissolved CH_2_Cl_2_ (2,5 L) in a 3 L round-bottomed flask under argon, with direct argon bubbling during one hour. Then methane sulfonic acid (MSA) (2.95 mL, 1.8 equiv.) was added and the reaction was stirred overnight. DDQ (30.6 g, 3 equiv.) was added, and the reaction mixture was stirred at room temperature for another hour. The reaction mixture was quenched by Et_3_N (2.5 mL) and evaporated under reduced pressure to give a black solid which was purified by silica gel column chromatography, using dichloromethane/cyclohexane (70/30) as the eluting solvent. The two least polar fractions to be collected together with impurities were the two expected atropisomers of porphyrin **4**, as shown by the MALDI-TOF monitoring. A second column chromatography with a gradient mixture dichloromethane/cyclohexane (from 70/30 to pure dichloromethane) allowed to isolate the two atropisomers as pure compounds with a global yield of 1,8 % (atropisomer αβ : 100 mg ; atropisomer αα : 317 mg). Atropisomer αβ: ^1^H NMR (CD_2_Cl_2_, 298 K, 500.14 MHz): δ 8.89 (4H, d, J = 4.80 Hz, βpyr), 8.84 (4H, d, J = 4.90 Hz, βpyr), 8,59–8,50 (2H, m, aro), 8.36–8,29 (2H, m, aro), 8.09 (2H, td, J = 7.80 Hz, J = 1.71 Hz, aro), 8.06 (2H, td, J = 7.68 Hz, J = 1.61 Hz, aro), -2.73 (2H, s, NH_int_). ^19^F NMR (CD_2_Cl_2_, 300 K, 470.52 MHz): δ -137.43 (4F, dd, J = 23.19 Hz, J = 7.88 Hz, F*_o_*), -153.13 (2F, t, J = 20.70 Hz, F*_p_*), -162.61 – -162.78 (4F, m, F*_m_*). UV-vis (CHCl_3_): *λ*/nm (10^-3^ *ε*, dm^3^.mol^-1^.cm^-1^): 416 (212.1), 511 (16.9), 544 (3.7), 590 (5.3), 644 (1.3). Atropisomer αα: ^1^H NMR (CDCl_3_, 300 K, 500.14 MHz): δ 8.80 (4H, d, J = 4.80 Hz, βpyr), 8.75 (4H, d, J = 4.90 Hz, βpyr), 8.51 (2H, dd, J = 8.10 Hz, J = 1.60 Hz, aro), 8.23 (2H, dd, J = 7.30 Hz, J = 1.70 Hz, aro), 8.01 (2H, td, J = 7.84 Hz, J = 1.62 Hz, aro), 7.97 (2H, td, J = 7.28 Hz, J = 1.68 Hz, aro), -2.70 (2H, s, NH_int_), UV-vis (CHCl_3_): *λ*/nm (10^-3^ *ε*, dm^3^.mol^-1^.cm^-1^): 416 (203.3), 511 (17.1), 544 (3.5), 590 (5.4), 644 (1.4).

**b) α-5,15-bis-(2-aminophenyl)-10,20-bis-(pentafluorophenyl)-porphyrin** **4**. αβ and αα atropisomers of the dinitroporphyrin **5** (1.13 mmol, 1 g) were dissolved in a mixture of concentrated HCl (94 mL) and EtOH (30 mL), a 1 L conical flask. SnCl_2_.2H_2_O (3.05 g, 12 equiv.) was added in one portion to the mixture. The resulting green solution was stirred for 2 days at RT. After completion of the reaction (monitored by MALDI and TLC), the solution was neutralized with aqueous KOH solution at 0 °C. The resulting violet solution was washed several times with water and the resulting porphyrin was extracted with CHCl_3_. The organic layers were collected and dried over MgSO_4_.

After evaporation of the solvent, the compound was dissolved in minimum amount of CH_2_Cl_2_ and purified by column chromatography with a gradient mixture dichloromethane/cyclohexane (from 80/20 to pure dichloromethane). It allowed to isolate the two atropisomers as pure compounds with a global yield of 66 % (atropisomer αβ : 253 mg ; atropisomer αα : 288 mg). Atropisomer αβ: ^1^H NMR (CDCl_3_, 300 K, 500.13 MHz): δ 9.04 (4H, d, J = 4.70 Hz, βpyr), 8.83 (4H, d, J = 4.80 Hz, βpyr), 7.88 (2H, d, J = 7.40 Hz, aro), 7.66 (2H, t, J = 7.80 Hz, aro), 7.22 (2H, t, J = 7.40 Hz, aro), 7.17 (2H, d, J = 8.20 Hz, aro), 3.59 (4H, s, NH_2_), -2.75 (2H, s, NH_int_). UV-vis (CHCl_3_): *λ*/nm (10^-3^ *ε*, dm^3^.mol^-1^.cm^-1^): 415 (297.2), 511 (26.9), 546 (9.5), 587 (10.3), 643 (5.3). Atropisomer αα: ^1^H NMR (CD_2_Cl_2_, 300 K, 500.13 MHz): δ 9.06 (4H, d, J = 4.80 Hz, βpyr), 8.85 (4H, d, J = 4.80 Hz, βpyr), 7.93 (2H, dd, J = 7.50 Hz, J = 1.50 Hz, aro), 7.66 (2H, td, J = 7.80 Hz, J = 1.60 Hz, aro), 7.23 (2H, t, J = 7.40 Hz, aro), 7.16 (2H, d, J = 8.20 Hz), 3.56 (4H, s, NH_2_), -2.73 (2H, s, NH_int_). ^19^F NMR (CD_2_Cl_2_, 300 K, 470.52 MHz): δ -136.43 (4F, dd, J = 23.80 Hz, J = 8.10 Hz, F*_o_*), -152.12 (2F, t, J = 20.70 Hz, F*_p_*), -161.73 – -162.06 (4F, m, F*_m_*). UV-vis (CHCl_3_): *λ*/nm (10^-3^ *ε*, dm^3^. mol^-1^.cm^-1^): 415 (264.9), 511 (24.8), 546 (8.7), 587 (9.2), 646 (6.9).

**c) α-5,15-bis-(2-[{3-chloromethyl}benzoylamido]-phenyl)-10,20-bis-(pentafluorophenyl)-porphyrin 3**. A 500 mL two neck round bottom flask equipped with a stirrer and cooled in an ice bath was charged with porphyrin **4** (atropisomer αα, 0.40 mmol, 353 mg), dry THF (30 mL) and NEt_3_ (330 μL, 6 equiv.). 3-(chloromethyl)benzoyl chloride (170 μL, 3 equiv.) was then added dropwise under argon atmosphere. The reaction mixture was allowed to stir 1h at 0 °C and then two hours warming at room temperature. Then the reaction was quenched by water and the organic layer was separated. The solvent was removed under vacuum. The resulting solid was dissolved in a mixture of dichloromethane/cyclohexane (DCM/C_6_H_12_: 50/50) and loaded on a silica gel chromatography column packed with the same mixture. The polarity of the mixture was increased up to 90/10 in DCM/C_6_H_12_ to elute the expected compound (quantitative yield). ^1^H NMR (CDCl_3_, 298 K, 400.16 MHz): δ 9.03 (4H, d, J = 4.90 Hz, βpyr), 8.93–8.86 (6H, m, βpyr + aro), 8.18 (2H, dd, J = 8.06 Hz, J = 1.60 Hz, aro), 7.97 (2H, td, J = 8.50 Hz, J = 1.60 Hz, aro), 7.66 (2H, td, J = 7.60 Hz, J = 1.30 Hz, aro), 7.51 (2H, s, NHCO), 6.82–6.72 (2H, m, aro), 6.53–6.45 (6H, m, aro), 3.46 (4H, s, CH_2_ bz), -2.68 (2H, s, NH_int_). ^19^F NMR (CDCl_3_, 300 K, 376.47 MHz): δ -136.78 (2F, dd, J = 24.00 Hz, J = 8.70 Hz, F*_o_*), -137.27 (2F, dd, J = 24.10 Hz, J = 8.60 Hz, F*_o’_*), -151.30 (2F, t, J = 20.70 Hz, F*_p_*), -161.17 – -161.39 (4F, m, F*_m_*). UV-vis (CHCl_3_): *λ*/nm (10^-3^ *ε*, dm^3^.mol^‑1^.cm^-1^): 419 (255.4), 512 (16.7), 548 (2.9), 587 (5.2), 642 (1.3).

**d) α-5,15-bis-({2,2-(3,3-[2,2-(diethoxycarbonyl)propane-1,3-diyl]-dibenzoyl-amido]-diphenyl)-10,20-bis-(pentafluorophenyl)-porphyrin 2**. Sodium metal (100 mg, 12 equiv.) was added to the absolute alcohol (20 mL) in a small round bottom flask and stirred for few minutes until the complete consumption of Na. Diethyl malonate (554 μL, 10 equiv.) was added to this solution at room temperature and stirred for half an hour. The resulting mixture was added to a solution of porphyrin **3** (0.365 mmol, 413 mg, 1 equiv.) in CH_2_Cl_2_ (65 mL) and the solution was turned immediately from violet to green. After 2h of stirring the reaction was quenched by H_2_O, the organic layer was separated and removed under vacuum. The desired product was purified on a silica gel chromatography column eluted with a mixture dichloromethane/cyclohexane (70/30). The expected compound was obtained in 88% yield (390 mg). ^1^H NMR (CDCl_3_, 300 K, 500.13 MHz): δ 9.18 (2H, dd, J = 8.50 Hz, J = 1.20 Hz, aro), 8.99 (4H, d, J = 4.80 Hz, βpyr), 8.86 (4H, d, J = 4.80 Hz, βpyr), 8.09 (2H, s, NHCO), 8.00 (2H, dd, 2H, J = 7.40 Hz, J = 1.60 Hz, aro), 7.94 (2H, td, J = 7.50 Hz, J = 1.60 Hz, aro), 7.71 (2H, dt, J = 7.90 Hz, J = 1.40 Hz, aro), 7.57 (2H, td, J = 7.50 Hz, J = 1.30 Hz, aro), 6.99 (2H, t, J = 7.70 Hz, aro), 6.76 (2H, dt, J = 7.60 Hz, J = 1.40 Hz, aro), 5.05 (2H, t, J = 1.80 Hz, aro), 1.80 (4H, s, CH_2_ bz), 0.94 (4H, bs, CH_2_), -0.73 (6H, s, CH3), -2.52 (2H, s, NH_int_). ^13^C NMR (CDCl_3_, 300 K, 125.77 MHz): δ 167.53, 164.52, 138.76, 136.39, 135.17, 133.49, 132.74, 130.38, 130.21, 128.36, 127.43, 125.65, 123.07, 120.15, 115.56, 103.07, 60.25, 58.47, 42.34, 29.72, 26.93, 11.67. ^19^F NMR (CDCl_3_, 300 K, 470.52 MHz): δ -136.47 (2F, dd, J = 23.00 Hz, J = 8.40 Hz, F*_o_*), -137.62 (2F, dd, J = 24.10 Hz, J = 8.30 Hz, F*_o’_*), -151.37 (2F, t, J = 20.70 Hz, F*_p_*), -161.20 (2F, td, J = 22.30 Hz, J = 8.50 Hz, F*_m_*), -161.39 (2F, td, J = 22.30 Hz, J = 8.50 Hz, F*_m’_*). ESI-HRMS: calcd m/z = 1217.3079 [M+H]^+^ for C_67_H_43_N_6_O_6_F_10_, found 1217.3082. UV-vis (CHCl_3_): *λ*/nm (10^-3^ *ε*, dm^3^.mol^-1^.cm^-1^): 418 (221.8), 511 (15.4), 542 (2.7), 587 (5.2), 642 (1.1).

**e) α-5,15-bis-({{2,2-(3,3-[2,2-(dicarboxylic acid)propane-1,3-diyl]-dibenzoyl-amido]-diphenyl)-10,20-bis-( pentafluorophenyl)-porphyrin 1**. Boron tribromide as a molar solution in dichloromethane (16.4 mL, 50 equiv.) was added to compound **2** (0.54 mmol, 600 mg) was dissolved in DCM (100 mL). After 12 h of stirring at RT, the reaction was completed. The mixture was quenched by water. The precipitated compound was filtered and the green solid was washed with water at pH = 7. The product was purified by silica gel chromatography column and eluted with CHCl_2_/MeOH/AcOH (86/12/2). Yield: 80% (501 mg). ^1^H NMR (DMSO-d^6^, 300 K, 500.14 MHz): δ 17.70 (2H, bs, CO_2_H) 9.17 (4H, bs, βpyr), 9.03 (2H, d, NHCO), 8.88 (4H, d, J = 4.80 Hz, βpyr), 8.58 (2H, d, J = 8.40 Hz, aro), 8.13 (2H, d, J = 7.40 Hz, aro), 7.92 (2H, t, J = 7.90 Hz, aro), 7.67 (2H, t, J = 7.50 Hz, aro), 7.38 (2H, d, J = 7.70 Hz, aro), 6.97 (2H, t, J = 7.80 Hz, aro), 6.81 (2H, d, J = 7.50 Hz, aro), 4.91 (2H, bs, aro), 1.62 (2H, bs, CH_2_ bz), 1.20 (2H, bs, CH_2_ bz), -2.66 (2H, bs, NH_int_). Partial ^13^C NMR from 2D HSQC(DMSO-d^6^, 300 K, 125.77 MHz): δ 136.2, 132.0, 129.9, 128.1, 126.7, 126.0, 124.3, 123.6, 43.8, 29.4. ^19^F NMR (DMSO, 298 K, 376.47 MHz): δ -137.51 (2F, dd, J = 25.70 Hz, J = 7.50 Hz, F*_o_*), -139.62 (2F, dd, J = 25.20 Hz, J = 7.60 Hz, F*_o’_*), -154.20 (2F, t, J = 22.60 Hz, F*_p_*), -162.77 (2F, td, J = 24.20 Hz, J = 8.00 Hz, F*_m_*), -163.28 (2F, td, J = 24.20 Hz, J = 7.80 Hz, F*_m’_*). ESI-HRMS: calcd m/z = 1159.2307 [M-H]^-^ for C_63_H_33_N_6_O_6_F_10_, found 1159.2310. UV-vis (CHCl_3_): *λ*/nm (10^-3^ *ε*, dm^3^.mol^-1^.cm^-1^): 423 (292.6), 512 (12.9), 550 (5), 587 (3.9), 643 (1.2).

**f) α-5,15-bis-({{2,2-(3,3-[2-(carboxylic acid)propane-1,3-diyl]-dibenzoyl-amido]-diphenyl)-10,20-bis-(pentafluorophenyl)-porphyrin 7**. Porphyrin **1** (0.42 mmol, 490 mg) was dissolved in 60 mL of toluene and heated at 110 °C overnight. A TLC and MALDI-TOF monitoring showed that about 30% of the starting material remained. After evaporation of the solvent, the two products **7** and **1** were separated by column chromatography. Porphyrin **7** was eluted with 1% MeOH in DCM and obtained in 65 % yield (305 mg). ^1^H NMR (CDCl_3_, 300 K, 500.13 MHz): δ 9.10 (2H, d, J = 8.60 Hz, aro), 9.09 (2H, d, J = 4.75 Hz, βpyr), 8.97 (2H, d, J = 4.70 Hz, βpyr), 8.95 (2H, d, J = 4.70 Hz, βpyr), 8.05 (2H, dd, J = 7.50 Hz, J = 1.60 Hz, aro), 7.97 (2H, t, J = 7.80 Hz, aro), 7.67 (2H, d, NHCO), 7.64–7.57 (4H, m aro), 7.01 (2H, t, J = 7.70 Hz, aro), 6.66 (2H, d, J = 7.50 Hz, aro), 5.14 (2H, s, aro), 1.79 (2H, dd, J = 13.20 Hz, J = 3.50 Hz, CH_2_ bz), 1.26–1.19 (1H, m, CH), 1.02 (2H, t_app_, J = 12.20 Hz, CH_2_ bz), -2.76 (2H, bs, NH_int_). Partial ^13^C NMR from 2D HSQC (CDCl_3_, 300 K, 125.77 MHz): δ 134.5, 131.5, 130.6, 128.8, 126.8, 124.2, 123.2, 120.2, 47.8, 38.1. ^19^F NMR (CDCl_3_, 300 K, 470.52 MHz): δ -135.92 (1F, dd, J = 24.00 Hz, J = 8.30 Hz, F*_o_*), -136.69 (1F, dd, J = 24.00 Hz, J = 8.50 Hz, F*_o_*), -136.98 (2F, td, J = 26.90 Hz, J = 8.10 Hz, F*_o_*), -151.14 (1F, t, J = 20.80 Hz, F*_p_*), -151.32 (1F, t, J = 20.53 Hz, F*_p_*), -161.12 (1F, td, J = 22.56 Hz, J = 7.80 Hz, F*_m_*), -161.30 (1F, td, J = 22.28 Hz, J = 8.76 Hz, F*_m_*), -161.35 (1F, td, J = 21.32 Hz, J = 8.19 Hz, F*_m_*), -161.55 (1F, td, J = 22.28 Hz, J = 8.76 Hz, F*_m_*). ESI-HRMS: calcd m/z = 1139.2374 [M+Na]^+^ for C_62_H_34_N_6_O_4_F_10_Na, found 1139.2380. UV-vis (CHCl_3_): *λ*/nm (10^-3^ *ε*, dm^3^.mol^-1^.cm^-1^): 419 (256.2), 512 (15.9), 545 (3), 585 (5.4), 639 (1).

**g) Iron insertion**. A free-base solution of porphyrin **7** in THF in the presence of an excess of iron bromide and 2,6-lutidine was heated at reflux overnight inside a glove box. The resulting mixture was taken out of the glove box, washed with HCl (1M), and dried. **scFe** was eluted with a mixture of 0.2% of MeOH in CHCl3 (78%). The structure of the complex was confirmed by HRMS. **7Fe**: ESI-HRMS: calcd m/z = 1170.1669 [M+H]^+^ for C_62_H_32_N_6_O_4_F_10_Fe, found 1170.1668. UV-vis (CHCl_3_): *λ*/nm (10^-3^ *ε*, dm^3^.mol^-1^.cm^-1^): 412 (94.4), 499 (9.7), 559 (sh, 4.7), 624 (4.1).

# X-Ray Diffraction Crystallography

X-ray diffraction data for compound **sc-Fe** was collected using a VENTURE PHOTONIII CMOS Bruker diffractometer with Micro-focus IuS source Mo Kα radiation. Crystal was selected under a polarizing optical microscope and glued in paratone oil. Crystals were mounted on a CryoLoop (Hampton Research) with Paratone-N (Hampton Research) as cryoprotectant and then flashfrozen in a nitrogen-gas stream at 200 K. For compounds, the temperature of the crystal was maintained at the selected value by means of a 700+ series Cryostream cooling device to within an accuracy of ±1K. Data reduction was accomplished using SAINT V7.53a. The substantial redundancy in data allowed a semi-empirical absorption correction (SADABS V2.10) to be applied, on the basis of multiple measurements of equivalent reﬂections. The structures were solved by direct methods using SHELXS-97^[[2]](#endnote-2)^ and refined against *F*^2^ by full-matrix least-squares techniques using SHELXL-2018^[[3]](#endnote-3)^ with anisotropic displacement parameters for all non-hydrogen atoms. Hydrogen atoms were introduced into the calculations as a riding model with isotropic thermal parameters. All calculations were performed by using the Crystal Structure crystallographic software package WINGX.^[[4]](#endnote-4)^ The crystal data collection and refinement parameters are given in Table S1.

CCDC 2363623 contains the supplementary crystallographic data for this paper. These data can be obtained free of charge from the Cambridge Crystallographic Data Centre and Fachinformationszentrum Karlsruhe via http://www.ccdc.cam.ac.uk/structures/.

**Table S1.** Crystallographic data and structure refinement details of **sc-Fe**.

| **Compound** | **sc-Fe** |
| --- | --- |
| CCDC | 2363623 |
| Empirical Formula | C_62_ H_31_ F_10_ Fe N_6_ O_4_, 0.694(C_3_ H_6_ O), 1.011(C H_4_ O), 0.295(H_2_ O) |
| *M_r_* | 1247.79 |
| Crystal size, mm^3^ | 0.08 x 0.06 x 0.03 |
| Crystal system | monoclinic |
| Space group | *P* 2_1_/c |
| a, Å | 13.6780(5) |
| b, Å | 16.3677(6) |
| c, Å | 25.6007(9) |
| α, ° | 90 |
| β, ° | 95.510(2) |
| γ, ° | 90 |
| Cell volume, Å^3^ | 5704.9(4) |
| Z ; Z’ | 4 ; 2 |
| T, K | 200(1) |
| Radiation type ; wavelength Å | MoKα ; 0.71073 |
| F_000_ | 2545 |
| µ, mm^–1^ | 0.357 |
| range, ° | 1.946 - 26.456 |
| Reflection collected | 129 180 |
| Reflections unique | 11 728 |
| R_int_ | 0.0857 |
| GOF | 1.106 |
| Refl. obs. (*I*>2(*I*)) | 7 412 |
| Parameters ; Restraints | 795 ; 9 |
| wR_2_ (all data) ^b^ | 0.3269 |
| R_1_ value (*I*>2(*I*)) ^a^ | 0.1088 |
| Largest diff. peak and hole (e-.Å^-3^) | 1.093 ; -0.803 |


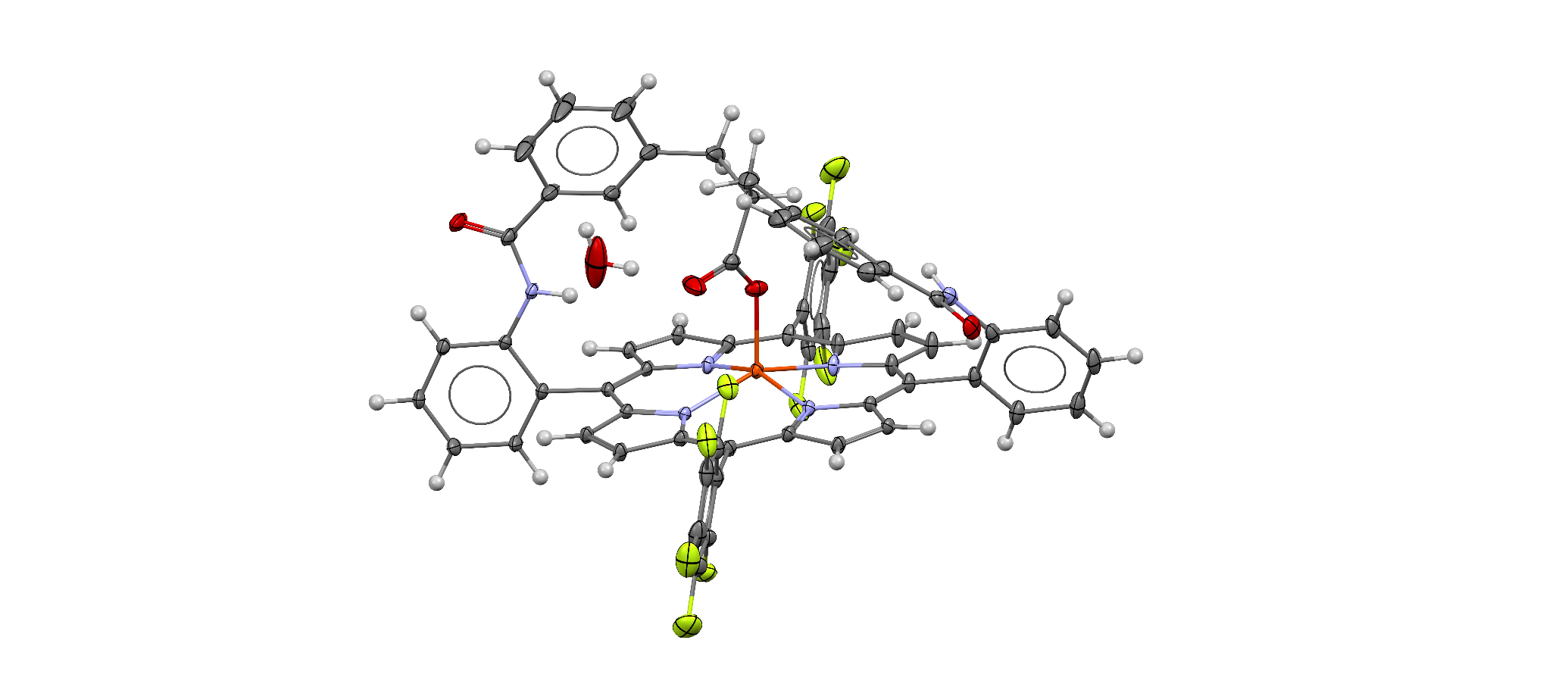


**Figure S2.** ORTEP drawing of compound **sc-Fe**. Thermal ellipsoids are shown at the 10% level. Solvent molecules were omitted for clarity except for the water molecule H-bonded to the carboxylate group.

# Electrochemical analysis

## Cyclic Voltammetry

### General methods and materials

Cyclic voltammetry (CV) measurements were recorded using a classic 3-electrode setup. The working electrode was a glassy carbon electrode with 0.3 cm diameter. Before each scan, the electrode was carefully polished with diamond paste (1 *µ*m), sonicated with ethanol, washed with ethanol and acetone, and dried. The counter electrode was a platinum wire and the reference was an aqueous Standard Calomel Electrode (SCE).

The measurements were carried out using a Metrohm Autolab PGSTAT320 potentiostat. Ohmic drop was compensated for each experiment. If not specified, 100 mV/s was used as the scan rate. The concentration of catalyst was 1 mM.

Bu_4_NPF_6_ (Aldrich, >99% purity) was used as supporting electrolyte at a concentration of 100 mM, and was recrystallized in absolute ethanol and dried overnight under high vacuum before use. Extra-dry dimethylformamide (DMF, 99.8%, AcroSeal, Thermo Scientific Chemicals) was used as solvents. Milli-Q water, deuterium oxide (D_2_O, Eurisotop, 99,90% D), 2,2,2-trifluoroethanol (TFE, Fluorochem, 99%) or phenol (PhOH, 99.5%, loose crystals, Thermo Scientific Chemicals) were used as proton sources.

Ferrocene (Fc, 99%, Thermo Scientific Chemicals) was used as internal standard to verify the stability of the reference electrode between two different experiments. With our aqueous SCE, we reported the following Fc/Fc^+^ standard potential shifts versus SCE, depending on the solvent used: in extra-dry DMF: *E°*(Fc/Fc^+^) = 450 mV, in a mixture of DMF:H_2_O (5M): *E°*(Fc/Fc^+^) = 400 mV (-50 mV *vs* dry DMF), in a mixture of DMF:TFE (3.5M): *E°*(Fc/Fc^+^) = 450 mV (0 mV *vs* dry DMF), in DMF with 2.4M PhOH: *E°*(Fc/Fc^+^) = 480 mV (+30 mV *vs* dry DMF), in extra-dry ACN: *E°*(Fc/Fc^+^) = 460 mV, and in a mixture of ACN:H_2_O (5M): *E°*(Fc/Fc^+^) = 410 mV (-50 mV *vs* dry ACN). Due to the very small shift of potential of Fc/Fc^+^ observed between ACN and DMF, we present here all our CVs in V *vs* SCE and we intentionally did not report all CVs *vs* Fc/Fc^+^ (it is known that *E°*(Fc/Fc^+^) is varying with the presence of acids in organic solvents).

Cyclic voltammograms are shown in normalized current (current is divided by $i_{p}^{\circ}$, intensity of the Fe^II^/Fe^I^ wave in Argon) versus the applied potential (V *vs* SCE). When adding H_2_O, TFE, or PhOH, the dilution of the solution due to the increase of the total volume of the solution was taken into account for the calculation of the concentration in proton source and for the normalization of the current ($i_{p}^{\circ}$).

Figure S3. Normalized cyclic voltammograms of sc-Fe in Ar-saturated dry DMF (black) and in Ar-saturated DMF + H_2_O (5 M) solvent mixture (violet).

Figure S4. Normalized cyclic voltammograms of sc-Fe^III/II^ reversible redox wave in Ar-saturated dry DMF (black), Ar-saturated DMF with 5 M H_2_O (violet), and CO_2_-saturated DMF with increasing amounts of H_2_O (dry DMF: green; light blue to deep blue: 0.55 M, 1.6 M, 2.6 M, 3.6 M, 4.1 M, 5 M). Ferrocene was added to the solution; *E°*(Fc/Fc^+^) = 450 mV *vs* SCE in dry DMF; *E°*(Fc/Fc^+^) = 400 mV *vs* SCE in DMF:H_2_O (5 M).

Figure S5. Normalized cyclic voltammograms of sc-Fe in CO_2_-saturated dry DMF (green) and in DMF with an increasing amount of H_2_O (light blue to deep blue: 2.6 M, 3.6 M, 4.1 M, and 5 M).

Figure S6. Normalized cyclic voltammograms of F_20_Fe in Ar-saturated dry DMF (black) and in Ar-saturated DMF + H_2_O (5 M) solvent mixture (violet).

Figure S7. Normalized cyclic voltammograms of F_20_Fe in CO_2_-saturated dry DMF (green) and in DMF with an increasing amount of H_2_O (light blue to deep blue: 2.6 M, 3.6 M, 4.1 M, and 5 M).

Table S2. Standard potential values for the three redox waves of sc-Fe and F_20_Fe, in argon-saturated DMF, dry or in presence of 5M H_2_O.

|  | *E°* /V *vs* SCE | *extra-dry DMF* | *H_2_O (5 M)* |
| --- | --- | --- | --- |
| **sc-Fe** | **Fe^III/II^** | -0.14 | -0.15 |
|  | **Fe^II/I^** | -1.18 | -1.14 |
|  | **Fe^I/0^** | -1.48 | -1.42 |
| **F_20_Fe** | **Fe^III/II^** | -0.02 | 0.03 |
|  | **Fe^II/I^** | -0.86 | -0.85 |
|  | **Fe^I/0^** | -1.37 | -1.36 |

Figure S8. Normalized cyclic voltammograms of sc-Fe in Ar-saturated dry DMF (black) and in Ar-saturated DMF with TFE (3.5 M) as proton source (violet).

Figure S9. Normalized cyclic voltammograms of sc-Fe in CO_2_-saturated dry DMF (green) and with an increasing amount of TFE (light orange to dark orange: 0.5 M, 1.0 M, 2 M, 3 M, and 3.5 M).

Figure S10. Normalized cyclic voltammograms of sc-Fe in Ar-saturated dry DMF (black) and in Ar-saturated DMF with PhOH (2.4 M) as proton source (violet).

Figure S11. Normalized cyclic voltammograms of sc-Fe in CO_2_-saturated dry DMF (green) and with an increasing amount of PhOH (light orange to dark orange: 0.5 M, 0.9 M, 1.7 M, and 2.4 M).

**Table S3.** Standard potential values for the three redox waves of sc-Fe, in argon-saturated DMF, dry or in presence of different proton sources. *Irreversible waves.

|  | *E°* /V *vs* SCE | *extra-dry DMF* | *H_2_O (5 M)* | *TFE (3.5 M)* | *PhOH (2.4 M)* |
| --- | --- | --- | --- | --- | --- |
| **sc-Fe** | **Fe^III/II^** | -0.14 | -0.15 | -0.11 | -0.13 |
|  | **Fe^II/I^** | -1.18 | -1.14 | -1.06 | -1.08* |
|  | **Fe^I/0^** | -1.48 | -1.42 | -1.38* | -1.36* |

## Determination of *k*_cat_ (Foot-of-the-Wave Analysis)

In catalytic conditions, the reaction of CO_2_ reduction to CO involves several steps (depending on the catalyst structure, on the potential applied or on other experimental conditions), but they are kinetically equivalent to an overall reaction with an apparent rate constant named $k_{cat}$ (s^-1^). This apparent rate constant is therefore the rate determining step. For homogeneous catalysis, the substrate is diffusing on the surface of the electrode where the active catalyst reacts with the substrate and then is regenerated on the electrode, to form again the catalytically active form of the catalyst. In order to calculate the apparent catalytical rate constant based on the intensity measured by CV, the concentration profile of the active form of the catalyst has to be determined, by integrating the diffusion equation in the thin diffusion layer next to the electrode.^[[5]](#endnote-5)^ Savéant and coworkers then combined the concentration profile of the catalyst active form to the current density (from Nernst law) to obtain the following expressions of normalized intensity and Turnover Frequency (TOF):

$$\frac{i}{i_{p}^{\circ}}=\frac{2.24\sqrt{\frac{RT}{F\nu}2k_{cat}}}{1+exp\left[ \frac{F}{RT}\left( E-{E^{\circ}}_{cat} \right) \right]} TOF=\frac{k_{cat}}{1+exp\left[ \frac{F}{RT}\left( E-{E^{\circ}}_{cat} \right) \right]}$$

with $i_{p}^{\circ}$ : peak intensity of the catalyst under non-catalytical conditions (Argon)

$$i_{p}^{\circ}=FA\cdot0.446\cdot{C^{\circ}}_{cat}\sqrt{D_{cat}}\sqrt{\frac{F\nu}{RT}}$$

$R$ : universal gas constant ($8.31446 J\cdot{mol}^{-1}\cdot K^{-1}$)

$T$ : temperature ($K$)

$F$ : Faraday constant ($9.6485\times{10}^{4} C\cdot{mol}^{-1}$)

$\nu$ : scan rate ($V\cdot s^{-1}$)

${E^{\circ}}_{cat}$: standard potential of the catalyst’s active species (here it is Fe^I^/Fe^0^ under argon in DMF with the proton source)

$A$ : surface area of the working electrode (${cm}^{2}$)

${C^{\circ}}_{cat}$ : bulk concentration of the catalyst (here ${C^{\circ}}_{cat}=1mM)$

$D_{cat}$ : diffusion coefficient of the catalyst (${cm}^{2}\cdot s^{-1}$)

For fast catalytic processes, the current-potential plot is ideally a S-shaped curve showing a plateau in intensity ($i_{pl}$) at high overpotential, with:

$$i_{pl}=FS{C^{\circ}}_{cat}\sqrt{D_{cat}}\sqrt{{2k}_{cat}}$$

Therefore, combining this expression of $i_{pl}$ with the expression of $i_{p}^{\circ}$ gives a direct access to $k_{cat}$:

$$k_{cat}=\left( \frac{i_{pl}}{i_{p}^{\circ}} \right)^{2}\frac{1}{{2.24}^{2}}\frac{F\nu}{2RT}={TOF}_{max}$$

Unfortunately, due to diverse side-phenomena we cannot use this method because the current-potential curve is not showing a real plateau at low potentials. In some cases, the effect of these secondary phenomena can be lowered by increasing the scan rate of the CV in order to decrease the charge passed on the electrode. ^[[6]](#endnote-6),^^[[7]](#endnote-7)^ However, at high scan rate, it is necessary to scan to lower potentials values in order to reach a plateau in intensity, which can be problematic if other reactions are happening at low potentials (solvent wall or proton reduction). In our case, the CO_2_ reduction catalytic wave is followed by another redox event (below -1.6V *vs* SCE) which makes the calculation of $k_{cat}$ not possible this way.

Savéant and coworkers showed that in the cases where the current-potential response deviates from the S-shape exponential. By plotting $i/{i_{p}^{\circ}}=f\left( 1/\left( 1+\exp\left( 1+f\left( E-{E^{\circ}}_{cat} \right) \right) \right) \right)$, with $f=F/{RT}$, a straight line should be obtained in the ideal case, and in practice, deviations are observed at higher overpotentials. It is caused by the increase of the charge passed through the electrode, increasing the side-phenomena discussed above. For this linear regression, the $i/i_{p}^{\circ}$ was corrected by the capacitive current at the origin of the catalytic wave. By considering only the beginning of the foot-of-the-wave, we can determine the rate constant (using only the linear portion for $1/\left( 1+\exp\left( 1+f\left( E-{E^{\circ}}_{cat} \right) \right) \right)$, *i.e.,* the slope at the origin). The slope at low overpotential therefore corresponds to the slope of the ideal S-shaped current-potential response:

$$slope=2.24 \sqrt{\frac{RT}{F\nu}{2k}_{cat}} , k_{cat}=\left( \frac{slope}{2.24} \right)^{2}\frac{F\nu}{2RT}$$

For **sc-Fe** and experiments using TFE and PhOH as proton sources, the scan rate was kept at 100 mV/s. However, due to the proximity of the Fe^II^/Fe^I^ wave with the catalytic wave in DMF with water (and D_2_O), their separation was not good enough to perform the foot-of-the-wave analysis at 100 mV/s. For this reason, we performed CVs at 500 mV/s and obtained a better separation between the two waves, and a shape of the catalytic wave closer to the canonical S-shape, therefore enabling a more accurate determination of $k_{cat}$ based on the foot-of-the-wave analysis. Moreover, we repeated the experiments 3 times (for H_2_O and D_2_O) in order to have a more precise estimation of $k_{cat}$ and a statistical incertitude on these values.

$$\bar{slope}=\left( {slope}_{1}{+slope}_{2}{+slope}_{3} \right)/3 \bar{k_{cat}}= \left( \frac{\bar{slope}}{2.24} \right)^{2}\frac{F\nu}{2RT}$$

$$\Delta slope= \frac{1}{\sqrt{3}}\sqrt{\frac{1}{2}\left( \left( {slope}_{1}-\bar{slope} \right)^{2}+\left( {slope}_{2}-\bar{slope} \right)^{2}+\left( {slope}_{3}-\bar{slope} \right)^{2} \right)}$$

$$\Delta k= \frac{\partial k}{\partial\left( slope \right)}\cdot\Delta slope=2\cdot\bar{slope}\cdot\frac{F\nu}{{2.24}^{2}\cdot2RT}\cdot\Delta slope$$

with $\bar{slope}$ : mean value of the initial slope of $i/{i_{p}^{\circ}}=f\left( 1/\left( 1+\exp\left( 1+f\left( E-{E^{\circ}}_{cat} \right) \right) \right) \right)$

$\bar{k_{cat}}$ : mean value of the apparent catalytic rate constant ($s^{-1}$)

${slope}_{1, 2, 3}$ : the three different slopes obtained from three separate experiments

$\Delta slope$ : statistical incertitude on the slope measurement

$\Delta k$: statistical incertitude on the catalytic rate constant $k_{cat}$ ($k_{cat}= \bar{k_{cat}}\pm\Delta k$) ($s^{-1}$)


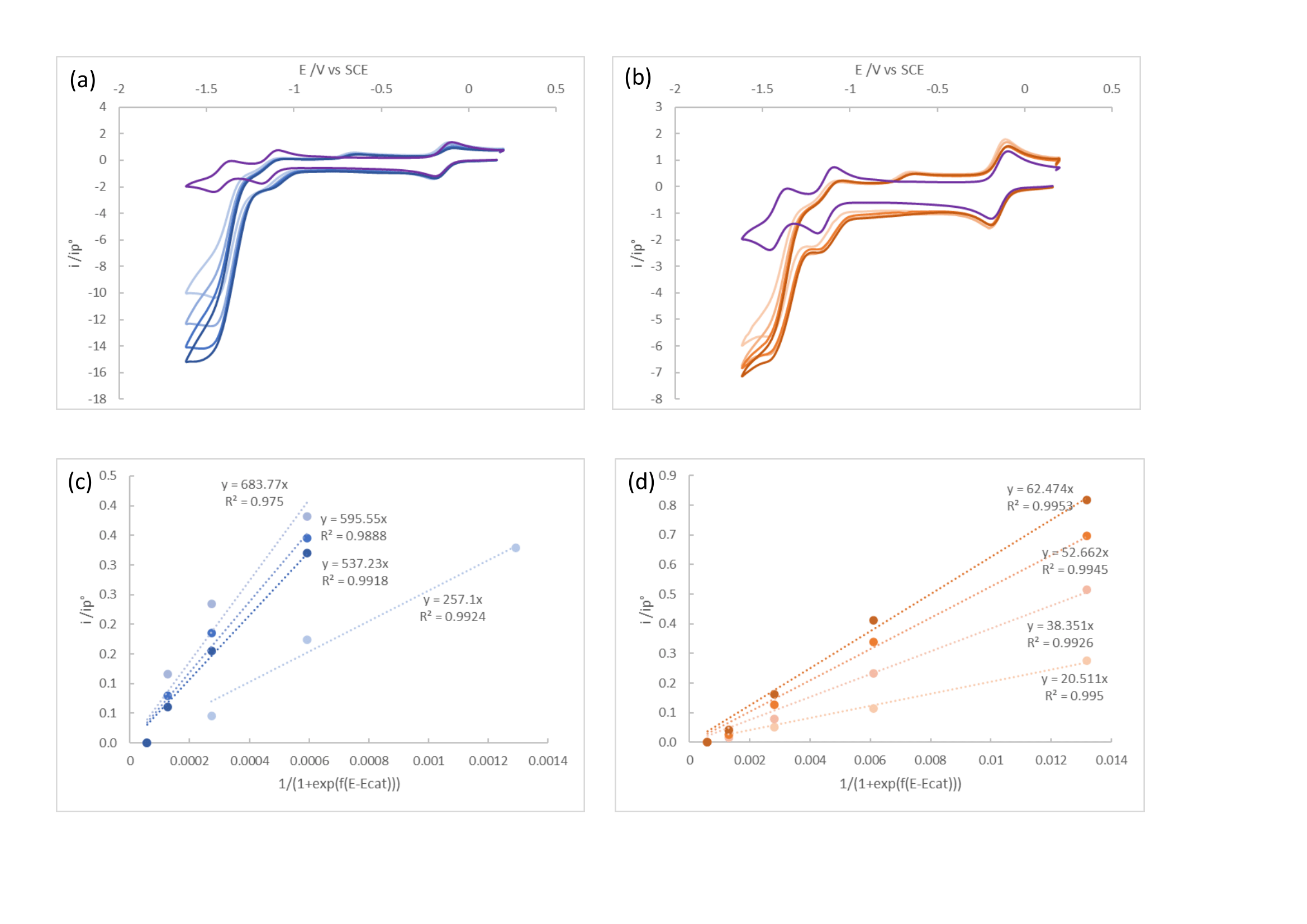
Figure S12. Normalized cyclic voltammograms of sc-Fe in CO_2_-saturated DMF with an increasing amount of H_2_O at 100 mV/s (a) (light blue to deep blue: 2.6 M, 3.6 M, 4.1 M, and 5 M; violet: Argon, 5 M H_2_O) and 500 mV/s (b) (light orange to dark orange: 2.6 M, 3.6 M, 4.1 M, and 5 M). Linearization of the current response $\boldsymbol{i}/{\boldsymbol{i}_{\boldsymbol{p}}\boldsymbol{^{\circ}}}\boldsymbol{=}\boldsymbol{f}\left( \boldsymbol{1}/\left( \boldsymbol{1+}\exp\left( \boldsymbol{1+}\boldsymbol{f}\left( \boldsymbol{E}\boldsymbol{-}{\boldsymbol{E}\boldsymbol{^{\circ}}}_{\boldsymbol{cat}} \right) \right) \right) \right)$ at 100 mV/s (c) and 500 mV/s (d).

At 100 mV/s, the catalytic wave is merged with the previous wave, so that linearity is not obtained (and the determination of the onset of the wave and the slope at the origin is arbitrary). The experiments at 500 mV/s show a better correlation. These experiments were repeated thrice, the results are reported in Table S4 and Table S7 for details and incertitude values.

Figure S13. (left) Normalized cyclic voltammograms at 100 mV/s of Fe_20_Fe in CO_2_-saturated DMF with an increasing amount of H_2_O (2.6 M, 3.6 M, 4.1 M, and 5 M; violet: Argon, 5 M H_2_O). (right) Linearization of the current response $\boldsymbol{i}/{\boldsymbol{i}_{\boldsymbol{p}}\boldsymbol{^{\circ}}}\boldsymbol{=}\boldsymbol{f}\left( \boldsymbol{1}/\left( \boldsymbol{1+}\exp\left( \boldsymbol{1+}\boldsymbol{f}\left( \boldsymbol{E}\boldsymbol{-}{\boldsymbol{E}\boldsymbol{^{\circ}}}_{\boldsymbol{cat}} \right) \right) \right) \right)$. The results are reported in Table S4.

Table S4. Apparent catalytic rate constants ($\boldsymbol{k}_{\boldsymbol{cat}}\boldsymbol{/}\boldsymbol{s}^{\boldsymbol{-1}}$) for sc-Fe and F_20_Fe in CO_2_-saturated DMF with H_2_O at various concentrations. For sc-Fe with H_2_O, $\boldsymbol{k}_{\boldsymbol{cat}}$ is calculated from three experiments sets at *ν* = 500 mV/s (cf. Table S7 for details and incertitude calculation).

| *[H_2_O]* | *2.6 M* | *3.6 M* | *4.1 M* | *5 M* |
| --- | --- | --- | --- | --- |
| **sc-Fe** | $2.4\times{10}^{3}$ | $6.2\times{10}^{3}$ | $9.0\times{10}^{3}$ | $1.8\times{10}^{4}$ |
| **F_20_Fe** | $2.4$ | $2.6$ | $2.8$ | $2.5$ |

**Figure S14.** (left) Normalized cyclic voltammograms at 100 mV/s of **sc-Fe** in CO_2_-saturated DMF with an increasing amount of TFE (light orange to dark orange: 0.5 M, 1.0 M, 2.0 M, 3.0 M, 3.5 M; violet: Argon, 3.5 M TFE). (right) Linearization of the current response $\boldsymbol{i}/{\boldsymbol{i}_{\boldsymbol{p}}\boldsymbol{^{\circ}}}\boldsymbol{=}\boldsymbol{f}\left( \boldsymbol{1}/\left( \boldsymbol{1+}\exp\left( \boldsymbol{1+}\boldsymbol{f}\left( \boldsymbol{E}\boldsymbol{-}{\boldsymbol{E}\boldsymbol{^{\circ}}}_{\boldsymbol{cat}} \right) \right) \right) \right)$.

**Table S5.** Apparent catalytic rate constants ($\boldsymbol{k}_{\boldsymbol{cat}}\boldsymbol{/}\boldsymbol{s}^{\boldsymbol{-1}}$) for **sc-Fe** in CO_2_-saturated DMF at various concentrations of TFE.

| *[TFE]* | *0.5 M* | *1.0 M* | *2.0 M* | *3.0 M* | *3.5 M* |
| --- | --- | --- | --- | --- | --- |
| **sc-Fe** | $7.58$ | $6.58\times{10}^{1}$ | $3.34\times{10}^{1}$ | $9.39\times{10}^{2}$ | $1.39\times{10}^{3}$ |

Figure S15. (left) Normalized cyclic voltammograms at 100 mV/s of sc-Fe in CO_2_-saturated DMF with an increasing amount of PhOH (light orange to dark orange: 0.5 M, 0.9 M, 1.7 M, 2.4 M; violet: Argon, 2.4M PhOH). (right) Linearization of the current response $\boldsymbol{i}/{\boldsymbol{i}_{\boldsymbol{p}}\boldsymbol{^{\circ}}}\boldsymbol{=}\boldsymbol{f}\left( \boldsymbol{1}/\left( \boldsymbol{1+}\exp\left( \boldsymbol{1+}\boldsymbol{f}\left( \boldsymbol{E}\boldsymbol{-}{\boldsymbol{E}\boldsymbol{^{\circ}}}_{\boldsymbol{cat}} \right) \right) \right) \right)$.

Table S6. Apparent catalytic rate constants ($\boldsymbol{k}_{\boldsymbol{cat}}\boldsymbol{/}\boldsymbol{s}^{\boldsymbol{-1}}$) for sc-Fe in CO_2_-saturated DMF with PhOH at various concentrations.

| *[PhOH]* | *0.5 M* | *0.9 M* | *1.7 M* | *2.4 M* |
| --- | --- | --- | --- | --- |
| **sc-Fe** | $1.80$ | $9.55$ | $2.13\times{10}^{1}$ | $2.47\times{10}^{1}$ |

Figure S16. Log(TOF_max_) *vs* p*K*_a_ of the proton source in DMF (blue: H_2_O – $\boldsymbol{pKa=31.5}$, orange: TFE – $\boldsymbol{pKa=24}$, yellow: PhOH – $\boldsymbol{pKa=18.8}$), for sc-Fe.

## Kinetic Isotopic Effect (KIE)

Kinetic isotopic effect was calculated based on the mean values of $k_{cat}$ obtained in DMF with H_2_O and D_2_O. The incertitude on KIE was calculated by propagating the incertitude obtained on the $k_{cat}$ values:

$$\bar{KIE}= \left( \frac{\bar{k}_{cat, H_{2}O}}{\bar{k}_{cat, D_{2}O}} \right)$$

$$\Delta KIE= \sqrt{{\frac{\partial KIE}{\partial k_{cat, H_{2}O}}}^{2}{\Delta k_{cat, H_{2}O}}^{2}+{\frac{\partial KIE}{\partial k_{cat, D_{2}O}}}^{2}{\Delta k_{cat, D_{2}O}}^{2}}=\bar{KIE}\sqrt{\left( \frac{\Delta k_{cat, H_{2}O}}{\bar{k}_{cat, H_{2}O}} \right)^{2}+\left( \frac{\Delta k_{cat, D_{2}O}}{\bar{k}_{cat, D_{2}O}} \right)^{2}}$$

with $\bar{KIE} :$mean value of KIE

$\bar{k}_{cat, H_{2}O}$, $\bar{k}_{cat, D_{2}O}$: mean values of the apparent catalytic rate constants under H_2_O or D_2_O

$\Delta KIE$ : statistical incertitude on KIE ($KIE=\bar{KIE}\pm\Delta KIE$)


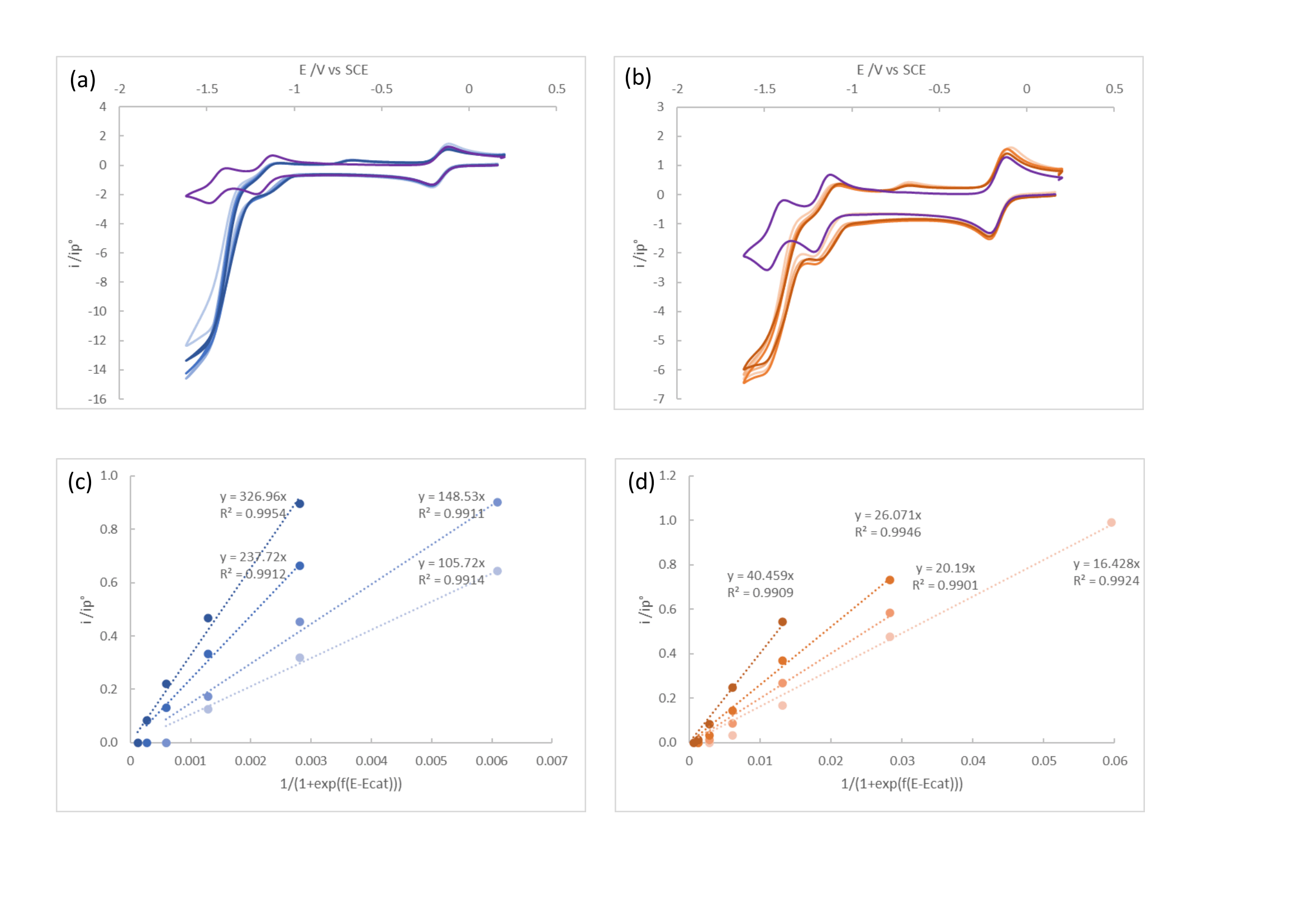
 $\Delta k_{cat, H_{2}O}$, $\Delta k_{cat, D_{2}O}$: statistical incertitude on the catalytic rate constants (H_2_O or D_2_O)

Figure S17. Normalized cyclic voltammograms of sc-Fe in CO_2_-saturated DMF with an increasing amount of D_2_O at 100 mV/s (a) (light blue to deep blue: 2.6 M, 3.6 M, 4.1 M, and 5 M; violet: Argon, 5 M D_2_O), and 500 mV/s (b) (light orange to dark orange: 2.6 M, 3.6 M, 4.1 M and 5 M). Linearization of the current response $\boldsymbol{i}/{\boldsymbol{i}_{\boldsymbol{p}}\boldsymbol{^{\circ}}}\boldsymbol{=}\boldsymbol{f}\left( \boldsymbol{1}/\left( \boldsymbol{1+}\exp\left( \boldsymbol{1+}\boldsymbol{f}\left( \boldsymbol{E}\boldsymbol{-}{\boldsymbol{E}\boldsymbol{^{\circ}}}_{\boldsymbol{cat}} \right) \right) \right) \right)$ at 100 mV/s (c) and 500 mV/s (d). These experiments were repeated thrice, the results are reported in Table S7 for details and incertitude values.

**Table S7.** Slopes obtained from the linearization of the foot-of-the-wave analysis on a set of 3 experiments for **sc-Fe** in CO_2_-saturated DMF at various concentrations of H_2_O or D_2_O at 500 mV/s, $\boldsymbol{k}_{\boldsymbol{cat}}$ (s^-1^) mean values, Kinetic Isotopic Effect (KIE), and associated statistical incertitude.

|  | [H_2_O] or [D_2_O] | 2.6 | 3.6 | 4.1 | 5 |
| --- | --- | --- | --- | --- | --- |
| H_2_O | ${slope}_{1, H_{2}O}$ | 20.511 | 38.351 | 52.662 | 62.474 |
|  | ${slope}_{2, H_{2}O}$ | 46.088 | 72.364 | 65.395 | 108.550 |
|  | ${slope}_{3, H_{2}O}$ | 37.912 | 58.557 | 86.127 | 118.52 |
|  | $\bar{{slope}_{H_{2}O}}$ | 34.8 | 56.4 | 68.1 | 96.5 |
|  | $\Delta{slope}_{H_{2}O}$ | 8 | 10 | 10 | 17 |
|  | $\bar{k}_{cat, H_{2}O}$ | **2.4E+3** | **6.2E+3** | **9.0E+3** | **1.8E+4** |
|  | $\Delta k_{cat, H_{2}O}$ | 1E+3 | 2E+3 | 3E+3 | 0.5E+4 |
| D_2_O | ${slope}_{1, D_{2}O}$ | n/a | 29.036 | 31.815 | 61.145 |
|  | ${slope}_{2, D_{2}O}$ | 16.428 | 20.190 | 26.071 | 40.459 |
|  | ${slope}_{3, D_{2}O}$ | 13.366 | 16.955 | 23.866 | 39.226 |
|  | $\bar{{slope}_{D_{2}O}}$ | 14.9 | 22.1 | 27.3 | 46.9 |
|  | $\Delta{slope}_{D_{2}O}$ | 1.1 | 3.6 | 2 | 7 |
|  | $\bar{k}_{cat, D_{2}O}$ | **4.3E+2** | **9.4E+2** | **1.4E+3** | **4.3E+3** |
|  | $\Delta k_{cat, D_{2}O}$ | 0.6E+2 | 3E+2 | 0.25E+2 | 1.3E+3 |
| **KIE** | $\bar{\boldsymbol{KIE}}$ | **5.5** | **6.5** | **6.2** | **4.2** |
|  | $\boldsymbol{\Delta KIE}$ | 2.5 | 3.1 | 2.1 | 2.0 |

## Catalyst Diffusion Coefficient

The diffusion coefficient of a catalyst per cm² of electrode surface can be determined by CV. We used glassy carbon as working electrode for both CV and electrolysis experiment.^[[8]](#footnote-1)^ Under non-catalytic conditions, the cathodic peak intensity $i_{p}$ is proportional to the square root of the scan rate, following Randles-Ševčík equation:

$$i_{p}=0.4463nFAC\left( \frac{nF\nu D_{cat}}{RT} \right)^{1/2}$$

with $n$ : number of electrons involved in the reaction (here $n=1$)

$F$ : Faraday constant ($9.6485\times{10}^{4} C\cdot{mol}^{-1}$)

$A$ : surface area of the working electrode (${cm}^{2}$)

$C$ : bulk concentration of the catalyst (here $C=1 mM)$

$\nu$ : scan rate ($V\cdot s^{-1}$).

$D_{cat}$ : diffusion coefficient of the catalyst (${cm}^{2}\cdot s^{-1}$)

$R$ : universal gas constant ($8.31446 J\cdot{mol}^{-1}\cdot K^{-1}$)

$T$ : temperature ($K$)

By plotting $i_{p}$ versus $\sqrt{\nu}$, and extracting its slope, we can calculate:

$$D_{cat}=\frac{{slope}^{2}RT}{\left( 0.4463nFAC \right)^{2}nF}$$

Figure S18. Linear regression of the cathodic peak intensity $\boldsymbol{i}_{\boldsymbol{p}}$ versus the square root of the scan rate $\sqrt{\boldsymbol{\nu}}$, for sc-Fe in DMF with 5 M H_2_O. Using the formula above: $\boldsymbol{D}_{\boldsymbol{cat}}\boldsymbol{=3.67\cdot}\boldsymbol{10}^{\boldsymbol{-7}}\boldsymbol{cm}^{\boldsymbol{2}}\boldsymbol{\cdot}\boldsymbol{s}^{\boldsymbol{-1}}$.

## Controlled potential electrolysis (CPE)

### Materials and setup

Bulk electrolysis experiments were undertaken using controlled potential for 2 hours with a Metrohm Autolab PGSTAT128N potentiostat, in a H-cell system with one compartment for the anolyte (6 mL) with the counter electrode, and the other compartment for the catholyte (7 mL) with the working electrode and the reference electrode (in a tube containing the electrolyte, separated with a frit), as shown on **Figure S19**. Bu_4_NPF_6_ (0.1 M) in DMF : H_2_O (10 : 1) was chosen as electrolyte. The catalyst was dissolved in 7 mL of electrolyte, at 0.5 mM. Both electrolytes were bubbled with CO_2_ for 20 min, the cell was sealed with rubber septa and the tightness was checked using the *µ*GC. A glassy carbon plate was used as working electrode, platinum mesh was used as counter electrode and a non-aqueous Ag/AgNO_3_ electrode was used as reference, for a better long-term stability in DMF. The conversion from Ag/AgNO_3_ to SCE was done by CV using the H-cell setup, before electrolysis. For the electrode used, we obtained a difference in respect to SCE of *E°*(Ag/AgNO_3_) = -250 mV *vs* SCE for the electrode used.

Gas products were detected by gas chromatography (*µ*GC, TraceGC Ultra, ThermoScientific). The following gas were detected at different retention times: H_2_ (54.9 s), O_2_ (62.5 s), N_2_ (70.4 s), CO (95.9 s). The amounts of H_2_ and CO were calibrated using the exact same setup, with the same volume of CO_2_-saturated electrolytes as used for the experiment (**Figure S20**).

Detection of liquid products was undertaken by NMR, on a *Bruker Avance 400 MHz*. A stock solution of D_2_O (D_2_O, Eurisotop, 99,90% D) with 200 mM phenol (99.5%, loose crystals, Thermo Scientific Chemicals) was prepared (94.1 mg phenol in 5 mL). The NMR tube was prepared by mixing 450 µL of the catholyte sample after electrolysis with 50 µL of the stock solution. The lock was done on the HDO peak and the reference of NMR spectrum was done on the aldehyde singlet peak of DMF (8.03 ppm). Characteristic peaks: DMF: 8.03 (s, 1H), 3.0 (s, 3H), 2.83 (s, 3H); H_2_O/HDO: 3.94 to 4.24 (s) depending on the amount of H_2_O; TBAPF_6_: 3.39-3.30 (m, 2H), 1.74 (dt, J = 15.6, 7.9 Hz, 2H), 1.43 (m, 2H), 1.00 (t, J = 7.3 Hz, 3H); PhOH : 7.21 (t, J = 7.7Hz, 2H), 6.87 ppm (d, J = 8.4Hz, 2H), 6.82 ppm (t, J = 7.3 Hz, 1H). No traces of CO_2_-reduction liquid products were observed in any CPE samples (methanol, formate, formaldehyde).


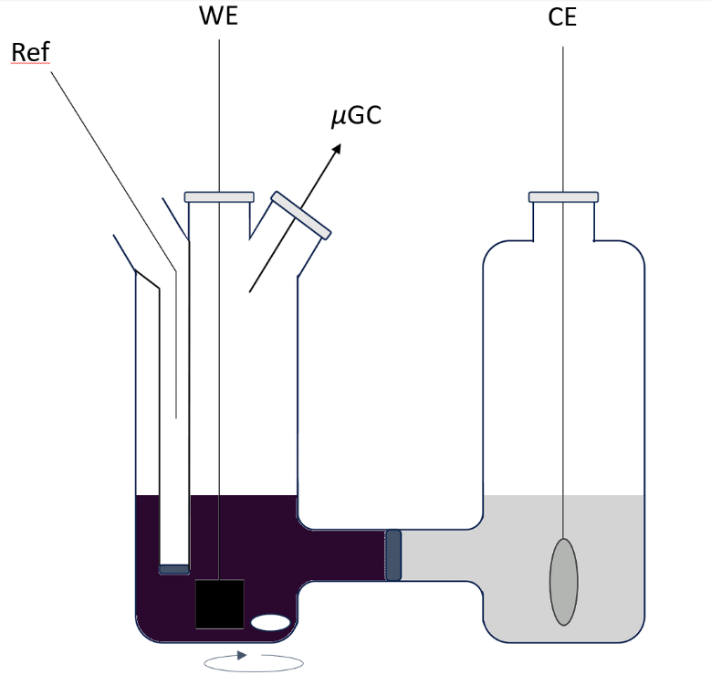


Figure S19 Sketch of the H-cell setup used for CPE.

Figure S20. Calibration curves of the H-cell used for CPE experiments: CO (left, retention time $\boldsymbol{t=95.9 s}$), H_2_ (right, retention time $\boldsymbol{t=55.6 s}$).

### Faradic Efficiency, selectivity and TON

The faradic efficiency (FE), selectivity (product molar ratio), TOF and TON were calculated from CPE experiments using the following expressions:

$${FE}_{CO}=2F\times n_{CO}/Q {FE}_{H_{2}}=2F\times n_{H_{2}}/Q$$

$$i=Q\times t$$

$$i_{CO}={FE}_{CO}\times i i_{H_{2}}={FE}_{H_{2}}\times i$$

$$k_{cat}=\frac{i_{CO}^{2}\left[ 1+\exp\left( \frac{F}{RT}\left( E_{el}-E_{cat} \right) \right) \right]^{2}}{2F^{2}A^{2}C_{cat}^{0}D_{cat}}$$

$$TOF= \frac{k_{cat}}{1+\exp\left( \frac{F}{RT}\left( E_{el}-E_{cat} \right) \right)} TON=TOF*t$$

with ${FE}_{CO, H_{2}}$ : Faradic efficiency for CO or H_2_ ($\%$)

$F$ : Faraday constant ($9.6485\times{10}^{4} C\cdot{mol}^{-1}$)

$n_{CO, H_{2}}$ : molar quantity of CO or H_2_ produced ($mol$)

$Q$ : charge passed for 2 hours of electrolysis ($C$)

$i$ : mean intensity of the electrolysis ($A$)

$t$ : electrolysis time ($s$)

$k_{cat}$ : apparent catalytic rate constant ($s^{-1}$)

$R$ : universal gas constant ($8.31446 J\cdot{mol}^{-1}\cdot K^{-1}$)

$T$ : temperature ($K$)

$E_{el}$ : applied electrolysis potential ($V$)

$E_{cat}$ : catalyst standard potential, $E^{\circ}\left( {Fe}^{I/0} \right)$, in $V$

$A$ : surface area of the working electrode (${cm}^{2}$) ^[[9]](#footnote-2)^

$C_{cat}^{0}$ : bulk concentration of the catalyst (here $C=1mM)$

$D_{cat}$ : diffusion coefficient of the catalyst (${cm}^{2}\cdot s^{-1}$)

$TOF$ : Turnover frequency of the catalyst ($s^{-1}$)

$TON$ : Turnover number, considering the molecules present in the electroactive diffusion layer

Additionally, if we consider all molecules of catalyst in the bulk, another value of TON can be calculated: $TON^{'}={n_{CO}}/{n_{cat}}$. This value is mostly relevant if we run the electrolysis until the current decreases to 0 A (which is rarely possible unless when using poorly stable catalysts or when operating at very high intensity), meaning that all molecules of catalyst in the entire solution participated and catalyzed CO_2_ reduction until they got fully degraded.

**Table S8.** Catalyst performances for **sc-Fe** calculated from CPE on glassy carbon plate electrode at two different potentials of electrolysis.

| $E_{el}$ /V *vs* SCE | ${FE}_{CO}$ | ${FE}_{H_{2}}$ | *Selectivity* $\left( CO:H_{2} \right)$ | $log\left( TOF/s^{-1} \right)$ | $TON$ | $TON^{'}$ |
| --- | --- | --- | --- | --- | --- | --- |
| **-1.55** | 80.4 % | 0.777 % | 99.0 : 0.99 | 2.2 | $1.15\times{10}^{6}$ | $5.6$ |
| **-1.40** | 71.5 % | 1.37 % | 98.1 : 1.88 | 2.4 | $2.00\times{10}^{6}$ | $3.1$ |

Figure S21. Current density ($\boldsymbol{j}$) profile *versus* time ($\boldsymbol{s}$) for sc-Fe during 2h electrolysis at $\boldsymbol{E}_{\boldsymbol{el}}\boldsymbol{=-1.55}\boldsymbol{V} \boldsymbol{vs} \boldsymbol{SCE}$ on a glassy carbon plate electrode ($\boldsymbol{A=0.8}\boldsymbol{cm}^{\boldsymbol{2}}$).

Figure S22. Current density ($\boldsymbol{j}$) profile *versus* time ($\boldsymbol{s}$) for sc-Fe during 2h electrolysis at $\boldsymbol{E}_{\boldsymbol{el}}\boldsymbol{=-1.40 V vs SCE}$ on a glassy carbon plate electrode ($\boldsymbol{A=0.84}\boldsymbol{cm}^{\boldsymbol{2}}$).

# UV-visible of sc-Fe

UV-visible spectra were recorded on a Agilent Cary 60 spectrophotometer in quartz cuvettes of 1 cm path length. The spectral window was set from 300 nm to 800 nm for all solvents. For each solvent, 4 solutions at 4 different concentrations were prepared.

Figure S23. Extinction coefficient of sc-Fe in DMF (mean value from 1.7 *µ*M, 3.4 *µ*M, and 6.8 *µ*M measurements).

Figure S24. Extinction coefficient of sc-Fe in DMF from 450 nm to 800 nm (Q-bands).

# Computational Details

**Methodology**

Density Functional Theory (DFT) calculations were carried out using Gaussian 16 revision B.01 package.^[[10]](#endnote-8)^ The geometries of the postulated intermediates were optimized without simplification. According to previous studies and the result of our computation, only the most stable spin states were considered in this study (see **Table S9**). The D3(BJ)^[[11]](#endnote-9)^ dispersion corrected B3LYP^[[12]](#endnote-10)^ hybrid functional was used in conjunction with the double-zeta def2-SVP^[[13]](#endnote-11)^ basis set. In addition, solvation correction for dimethylformamide were included using the SMD^[[14]](#endnote-12)^ polarizable conductor calculation model as implemented in Gaussian. Vibrational frequencies and thermal correction were computed at 298.15 K using the same level of theory. Single point calculations were performed on top of the geometry optimizations by enlarging the basis set to the valence triple-zeta with two sets of polarization functions def2-TZVPP. Transition state geometries were confirmed and verified as saddle points by the presence of a single imaginary frequency. These geometries were then linked to the corresponding saddle point minima of reactants and products via Intrinsic Reaction Coordinate (IRC) analysis. Cartesian coordinates of all intermediates are provided with their energies and spin distribution in a unique text file available as supplementary information material. The Non-Covalent Interaction (NCI) analysis was performed using NCIPlot^[[15]](#endnote-13)^ and the non-covalent regions were plot with VMD^[[16]](#endnote-14)^ and GNUPlot.

**Table S9.** Charge and spin states of the intermediates considered in this study

| **Intermediate** | **charge** | **Spin state** |
| --- | --- | --- |
| sc-Fe(III) | 0 | sextet |
| sc-Fe(II) | -1 | quintet |
| sc-Fe(I) | -2 | quadruplet |
| scH-Fe(II) | 0 | triplet |
| scH-Fe(I) | -1 | doublet |
| scH-Fe(III)-CO_2_ | -1 | doublet |
| scH-Fe(III)-CO_2_H | 0 | doublet |
| scH-Fe(II)-CO_2_H | -1 | singlet |
| scH-Fe(II)-CO | 0 | singlet |
| sc-Fe(III) | 0 | sextet |
| sc-Fe(II) | -1 | quintet |
| sc-Fe(I) | -2 | quadruplet |
| scH-Fe(II) | 0 | triplet |
| scH-Fe(I) | -1 | doublet |
| scH-Fe(III)-CO_2_ | -1 | doublet |
| scH-Fe(III)-CO_2_H | 0 | doublet |
| scH-Fe(II)-CO_2_H | -1 | singlet |
| scH-Fe(II)-CO | 0 | singlet |

**Redox potential**

Thermodynamic redox potentials were calculated according to the isodesmic method, using the **sc-Fe^(III/II)^** redox potential as a reference. For the latter the following relations were used and the calculated potential was corrected with respect to the experimental one (see Figure S25).

$$E^{\circ} = E - E_{SCE}$$

with $E=\frac{{G\left( \mathrm{ox} \right)}_{\text{dmf}} - {G\left( \mathrm{red} \right)}_{\text{dmf}}}{nF}$ and  *E*_SCE_ = 4.35 V ^[[17]](#endnote-15)^

**Proton-coupled electron transfer potential estimation**

The potential of PCET :

$\mathrm{ox}+ \mathrm{AH} + e^{-}\underset{\to}{E^{\circ}} redH+ A$

were calculated using the Nernst Equation:

$E_{PCET}= E^{\circ}-0.059\log\frac{\left[ A \right]}{\left[ AH \right]}$ where *AH* is the proton source, and *A* its conjugated base.

In the absence of CO_2_, the concentration of A (1.87 10^-12^ M) was derived from the initial concentration of the acid AH ([TFE]=3.5 M) and its pKa value in DMF (24). In the presence of CO_2_, we adapted a previously reported procedure.^^[[18]](#endnote-16)^^ The stronger acid in solution is H_2_CO_3_, coming from the hydration of CO_2_ in the presence of water (*K*_h_ = 1.7 × 10^-3^)^^[[19]](#endnote-17)^^. Given the concentration of water (5.05 M) and the solubility of CO_2_ in water and DMF (3.4 × 10^-2^ and 2.3 × 10^-1^ M respectively), the solubility of CO_2_ in the mixture was approximated to 2.1 × 10^-1^ M as the weighed sum of the concentration in each solvent. By neglecting the change of hydration equilibrium of CO_2_ and p*K*_a_ of H_2_CO_3_ in the solvent mixture (p*K*_a_^mix^≈ p*K*_a_^DMF^=6.4), the following concentrations were calculated: [H_2_CO_3_] = 3.3 × 10^-5^ M; [HCO_3_^-^] = 3.8 × 10^-6^ M.

*E_calc_ = –0.34V vs SCE corrected to –0.14V*

**Figure S25.** Structures of the high spin (S=5/2) **sc-Fe(III)** and the low spin open-shell (S=1) **sc-Fe(II)** species, taken as reference for the computation of the redox potentials using the isodesmic method.

**Figure S26.** Square scheme and thermodynamics data allowing to explain the 2^nd^ reduction process probed by CV from sc-Fe(II). Potential calculated *vs* SCE using the isodesmic method, E_PCET_ obtained from the Nernst Equation with 3.5M of TFE as proton source.

*Note that under catalytic conditions, the acid source is the carbonic acid (H_2_CO_3_) formed upon hydration of CO_2_ in water and the corresponding values are obtained instead: ∆G°_1_ = + 6.5 kcal/mol ; ∆G°_2_ = 1.0 kcal/mol ; E_PCET_ = -1.21 V.*

Trying to pull the carboxylic group from the first coordination sphere in **scH-Fe(I)** led to a more stable conformer with less steric hindrance around iron :

**Figure S27.** Unfolding of the diphenylpropyl strap allowed after the formation of **scH-Fe(I)** by PCET.

The deprotonation of this stabilized species is disfavored by 5.4 kcal/mol in a CO_2_-saturated DMF/H_2_O medium, thus allowing credence for participation of this intermediate in the catalytic process.

However, according to the Fe-H distance featured on this unfolded structure, some interaction between the Fe center and the carboxylic proton seemed to remain. To characterize the nature of this interaction the electronic structure of the complex was first analyzed. The global spin state of the scHFe(I) species is a doublet that results from a triplet iron ion antiferromagnetically coupled to the porphyrinyl radical. The dz^2^ orbital is doubly occupied with no covalent mixing with the orbitals of the hydroxyl group of the hanging ligand.

**Figure S28.** Detail of the electronic configuration of the doublet scFe(I) species with formal labelling and plots of the Fe 3d and Porphyrin Gouterman frontiers orbitals.

We then performed a NCI analysis which evidenced that the carboxylic group rests over the metal due to non-covalent interactions of the carboxylic proton with both the Fe and a pyrrolic N atom, with some possible hydrogen bonding character (blueish surfaces A in the figure below). In addition, the two methylene groups bridging the carboxylic moiety also establish Van der Waals interactions with the Fe porphyrin system (green surfaces B and C in the figure below), that may reinforce the former interaction.


**Figure S29.** NCI analysis in the area between the bridging ligand and the FeN_4_ moiety, a) 3D plot showing the whole **scFe(I)** molecule, b) zoom showing the 3 center Fe…H…N non covalent interaction (A) with Gradient isosurface s = 0.4 a.u. and color range +/- 4, c) 2D plot of the density gradient (s) against the sign of the second eigenvalue of the electron density Hessian matrix (sign(λ_2_)) multiplied by the electron density (ρ). Repulsive interaction appears in red (λ_2_>0), attractive in blue (λ_2_<0), weak in green (λ_2_≈0).

**Figure S30.** Attempts to model the coordination of CO_2_ at the nonfunctionalized face of **sc-Fe** catalyst

**Figure S31.** ***a-b***: Geometries computed for the Fe(I)-CO_2_ adducts with OH moiety of the strap pointing towards or away from the bound carbon dioxide (***a*** is 5.3 kcal/mol more stable than ***b***). ***c***: Possible geometry obtained with a water molecule intercalated; C-O distances are given in Angstrom, OCO angle in degree. ***d***: Natural charges (NPA) calculated at Fe and CO_2_, showing the influence of the strap and water on the charge transfer to the CO_2_ fragment.

**Figure S32.** Energetic profile and structures involved in the formation of the **Fe(I)-CO_2_** adducts from **sc-Fe(I)**, with or without a water molecule intercalated. The dot lines in the structures illustrate the possible H-bonding interactions, green lines are used to display the very long NH...O distances in the absence of water (no interaction). The geometries of the TS are in agreement with a late transition state (closer to the product) in the absence of water, and an early one in the presence of water (closer to the substrates).


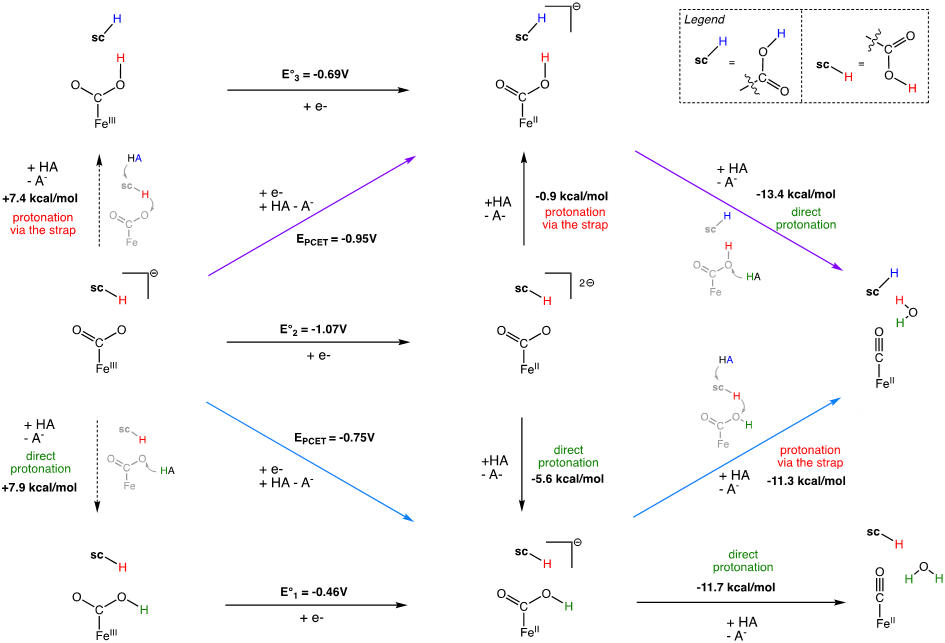


**Figure S33.** Thermodynamic pathways computed for the 1e^‒^, 2H^+^ reduction of the **scH-Fe(III)-CO_2_** species leading to the formation of Fe(II)-CO. Two accessible pathways involving a protonation via the pending carboxylic moiety are shown in color.

# Spectroscopic data (NMR and HRMS spectra)


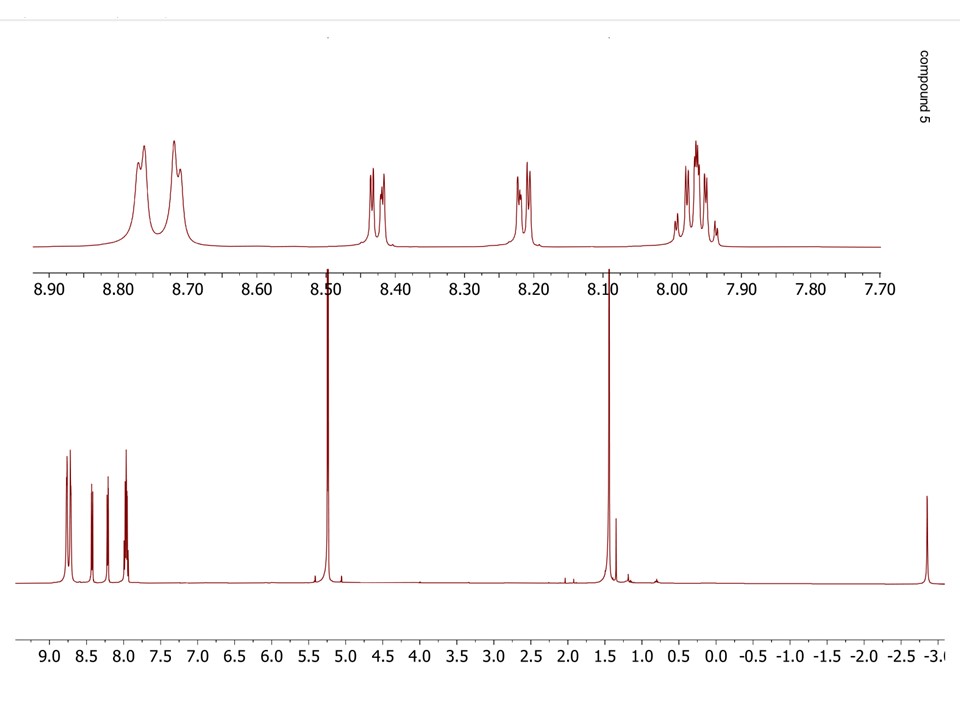


**Figure S34.** ^1^H NMR spectrum (500 MHz, CDCl_3_, 300 K) of porphyrin **5**.


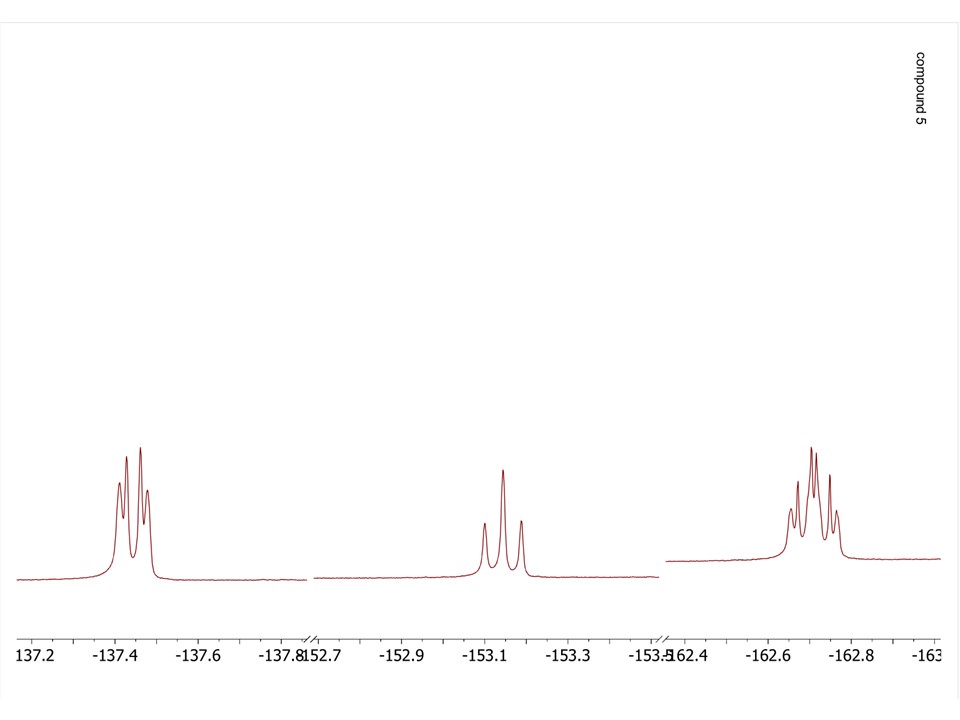


**Figure S35.** ^19^F NMR spectrum (376 MHz, CDCl_3_, 300 K) of porphyrin **5**.


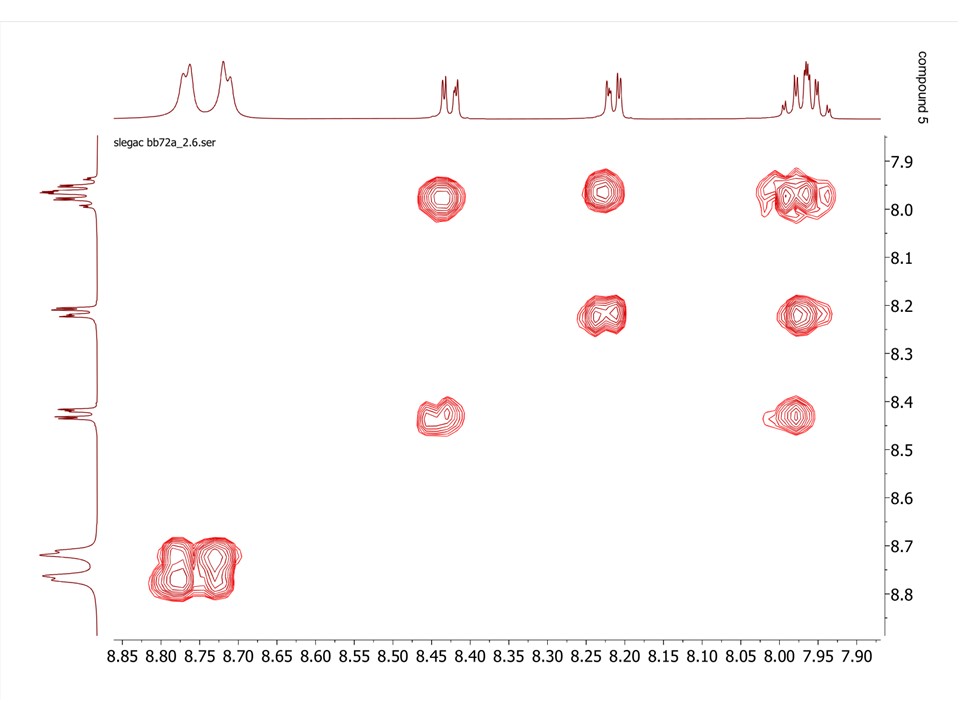


**Figure S36.** 2D COSY ^1^H NMR spectrum (500 MHz, CDCl_3_, 300 K) of porphyrin **5**.


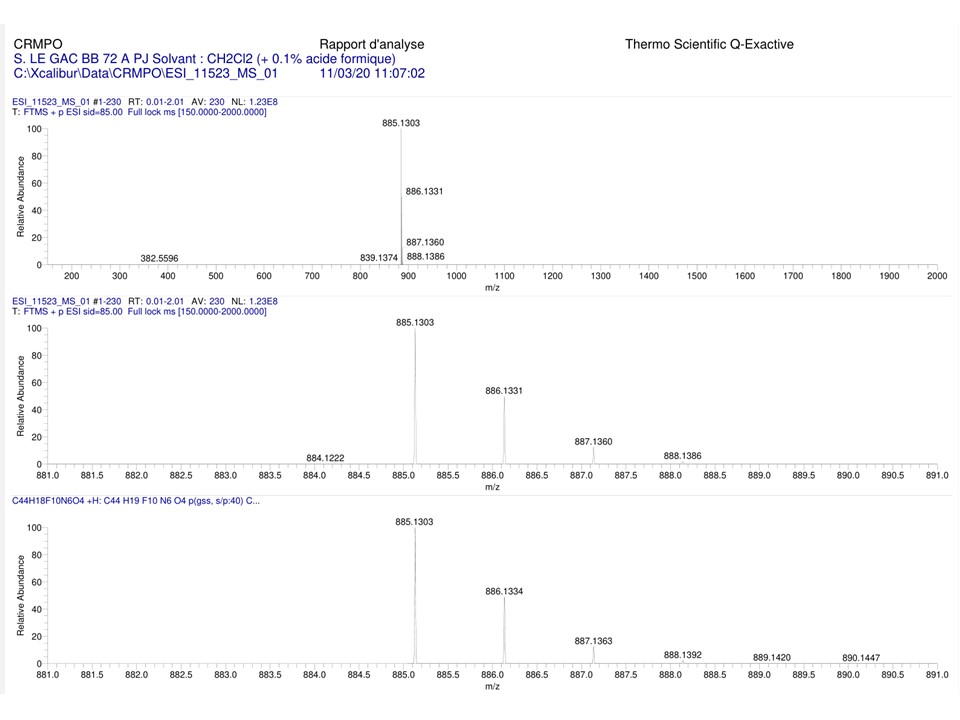


**Figure S37.** ESI-HRMS spectrum of **5**.


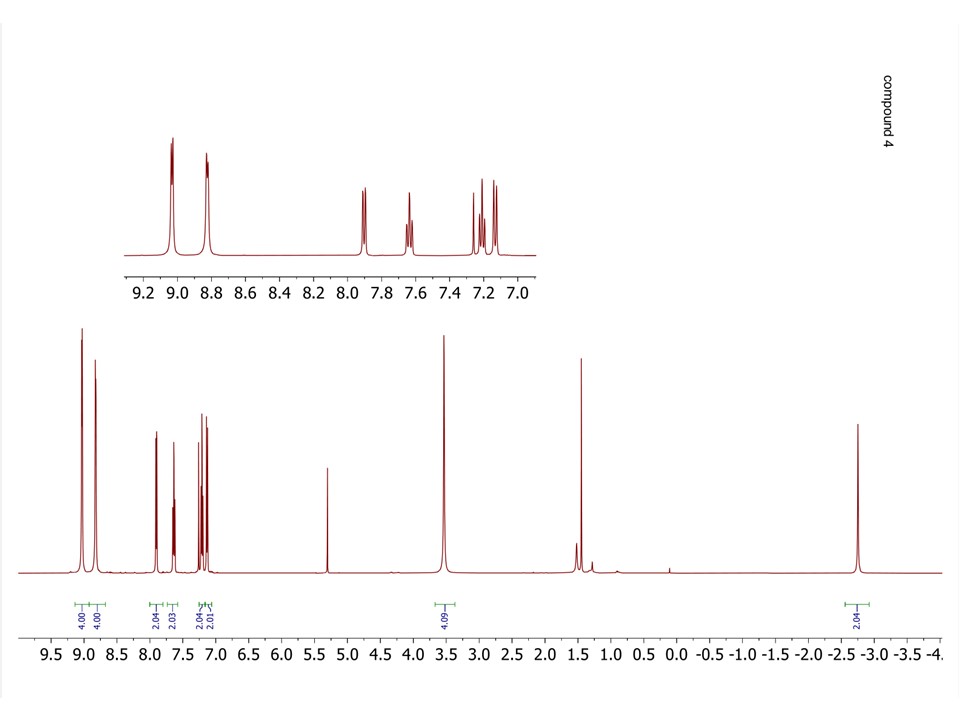


**Figure S38.** ^1^H NMR spectrum (500 MHz, CDCl_3_, 300 K) of porphyrin **4**.


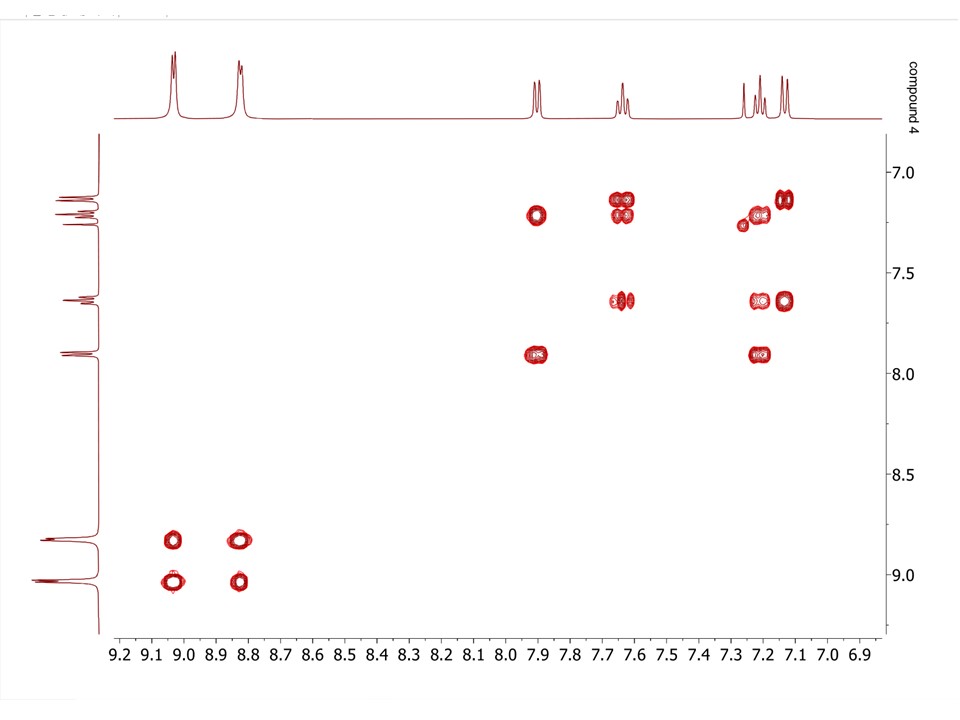


**Figure S39.** 2D COSY ^1^H NMR spectrum (500 MHz, CDCl_3_, 300 K) of porphyrin **4**.


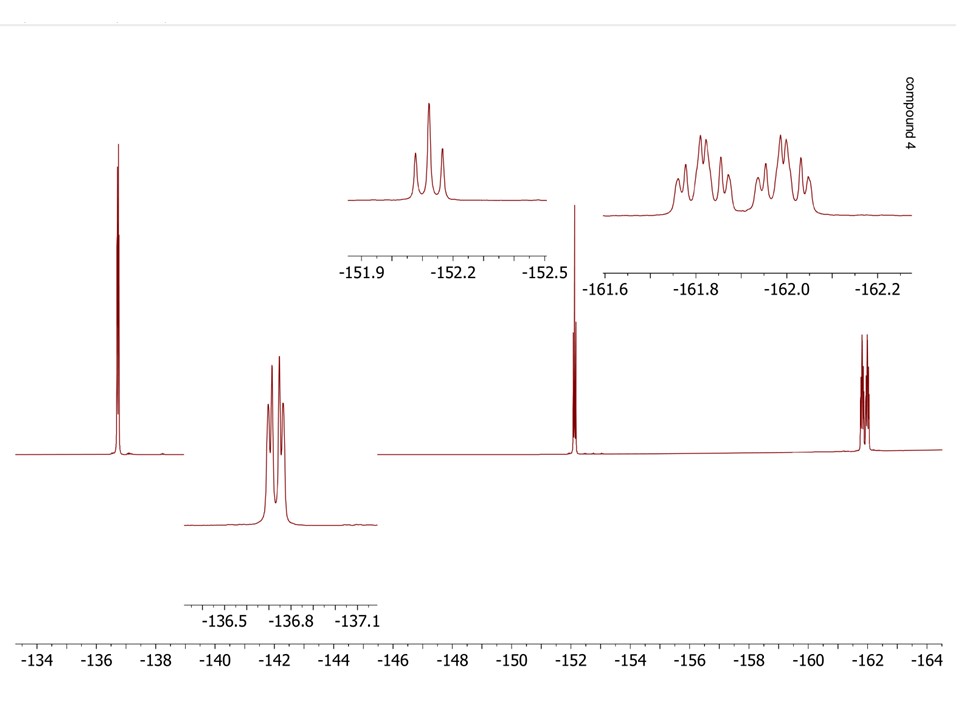


**Figure S40.** ^19^F NMR spectrum (376 MHz, CDCl_3_, 300 K) of porphyrin **4**.


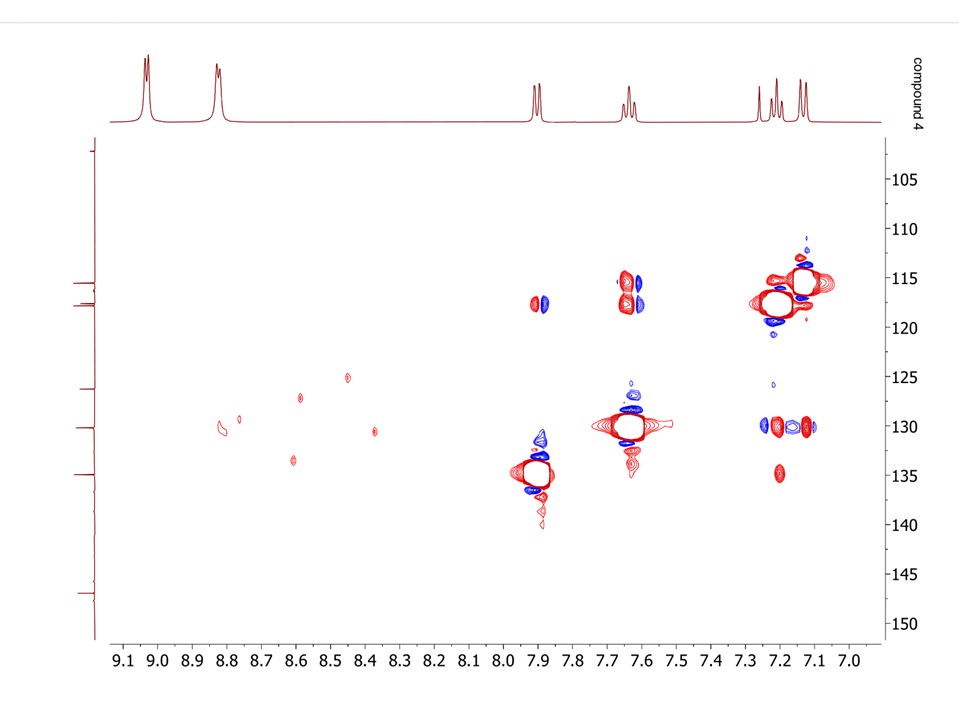


**Figure S41.** 2D HSQC ^1^H NMR spectrum (500 MHz, CDCl_3_, 300 K) of porphyrin **4**.


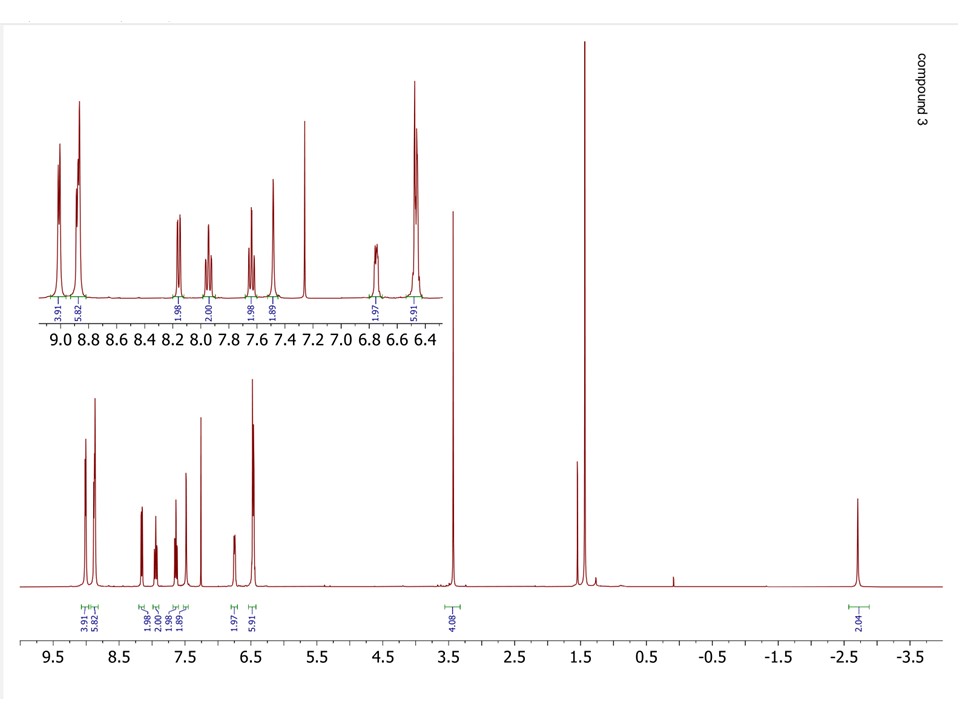


**Figure S42.** ^1^H NMR spectrum (500 MHz, CDCl_3_, 300 K) of porphyrin **3**.


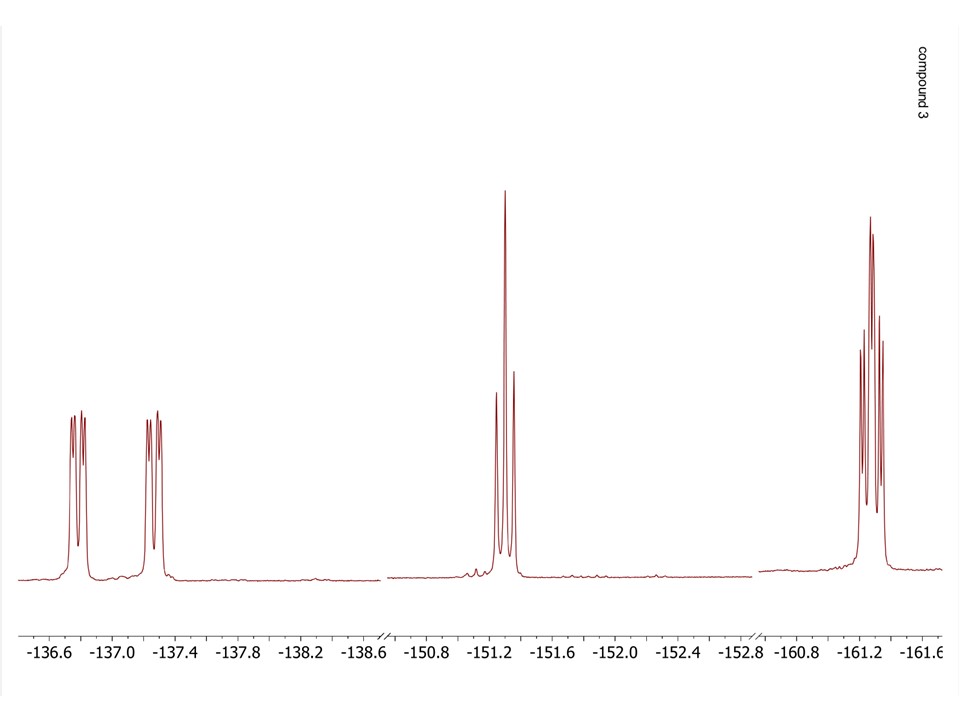


**Figure S43.** ^19^F NMR spectrum (376 MHz, CDCl_3_, 300 K) of porphyrin **3**.


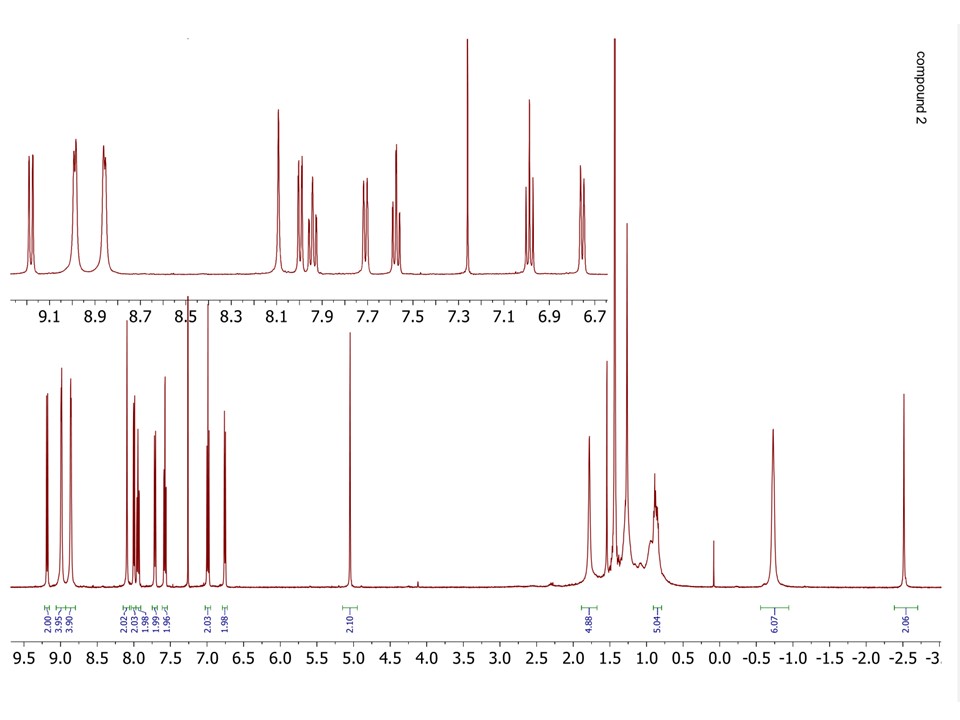


**Figure S44.** ^1^H NMR spectrum (500 MHz, CDCl_3_, 300 K) of porphyrin **2**.


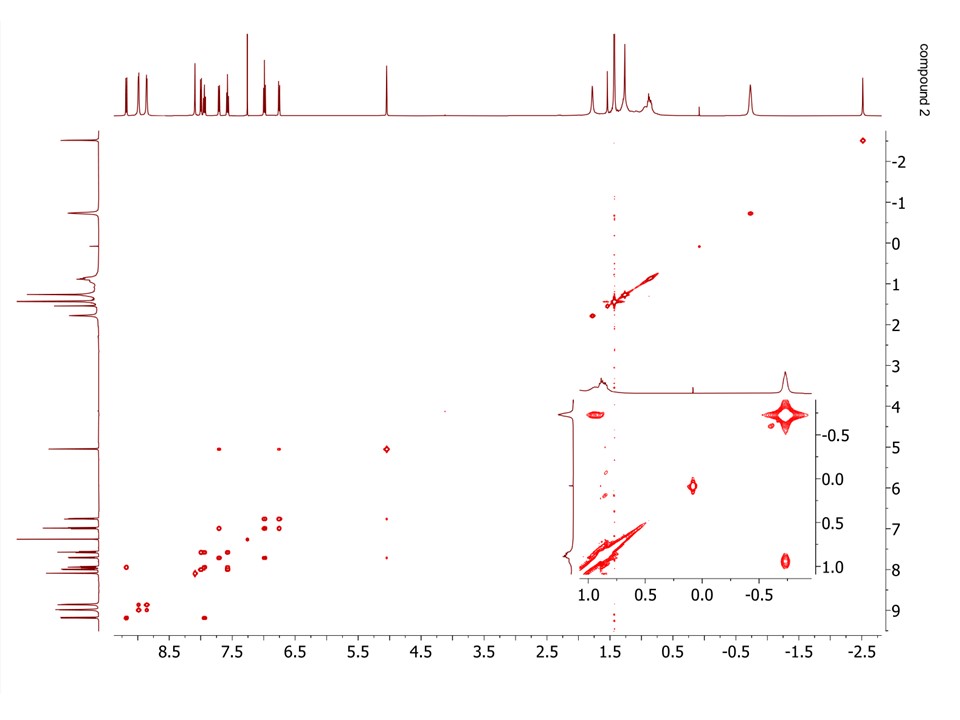


**Figure S45.** 2D COSY ^1^H NMR spectrum (500 MHz, CDCl_3_, 300 K) of porphyrin **2**.


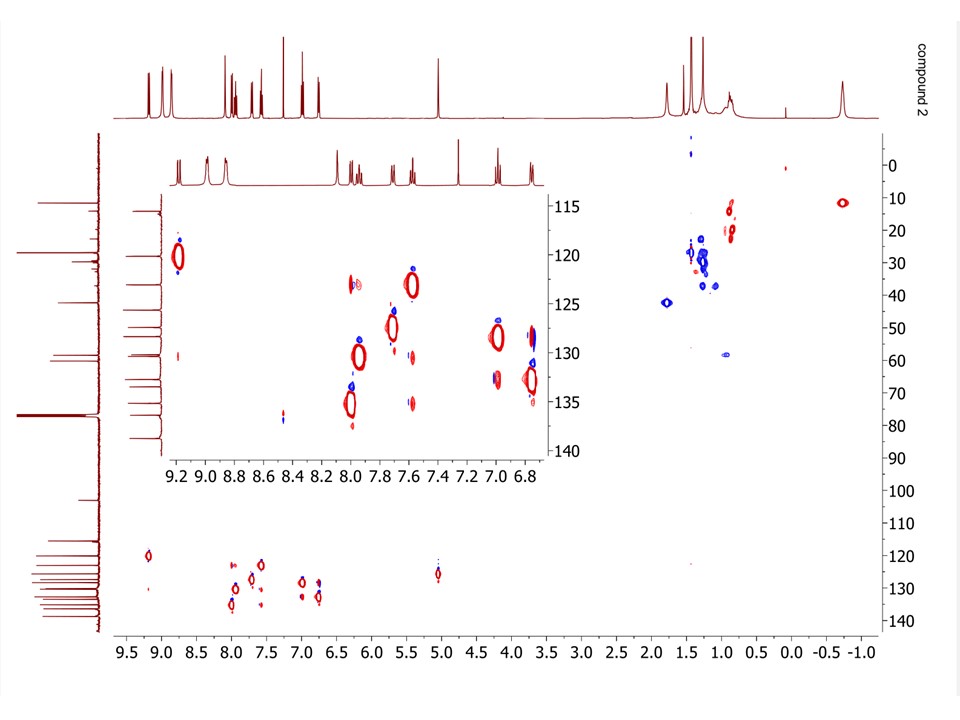


**Figure S46.** 2D HSQC ^1^H NMR spectrum (500 MHz, CDCl_3_, 300 K) of porphyrin **2**.


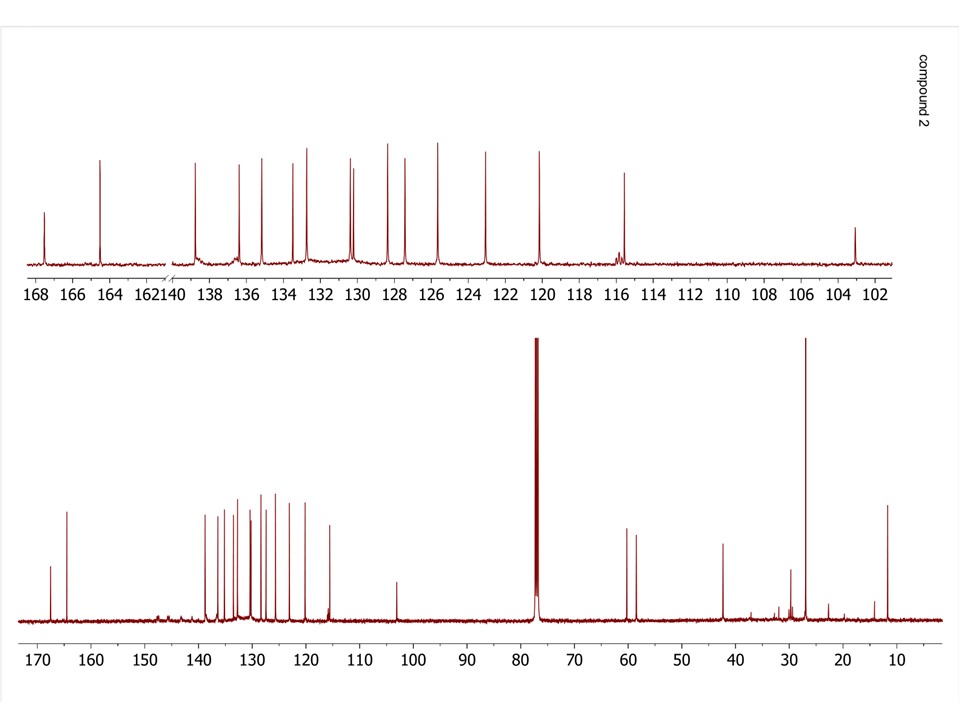


**Figure S47.** ^13^H NMR spectrum (125 MHz, CDCl_3_, 300 K) of porphyrin **2**.


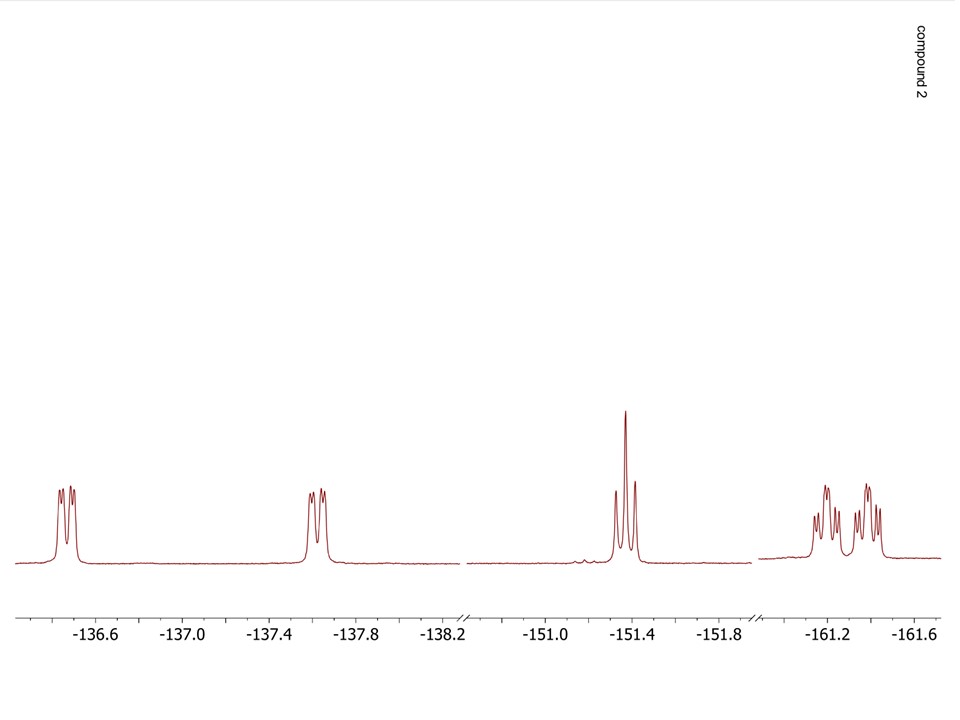


**Figure S48.** ^19^F NMR spectrum (376 MHz, CDCl_3_, 300 K) of porphyrin **2**.

**
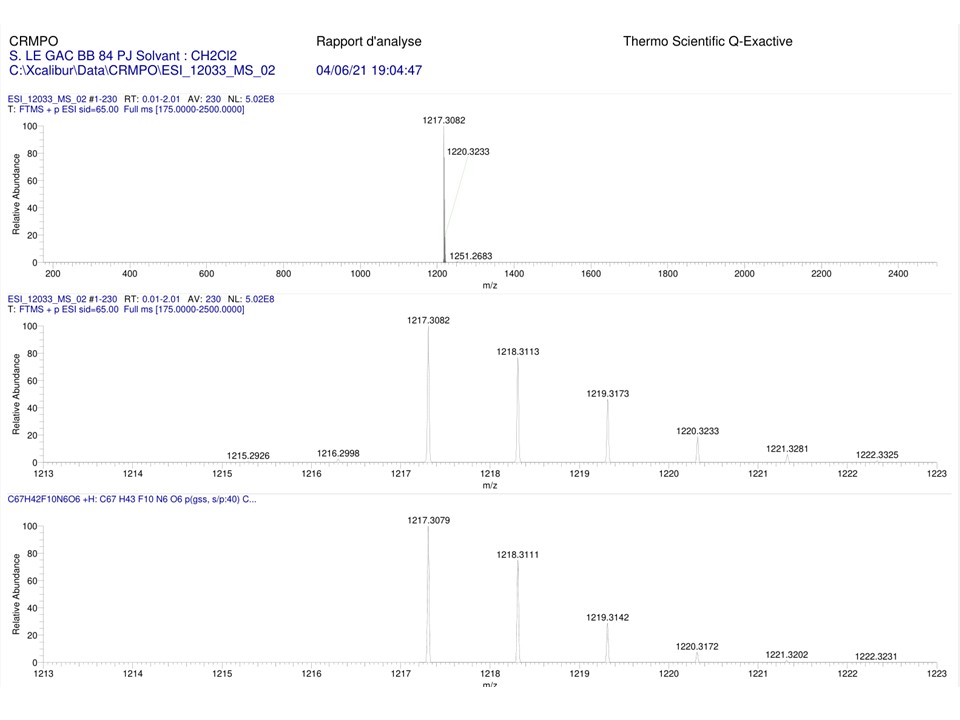
**

**Figure S49.** ESI-HRMS spectrum of **2**.


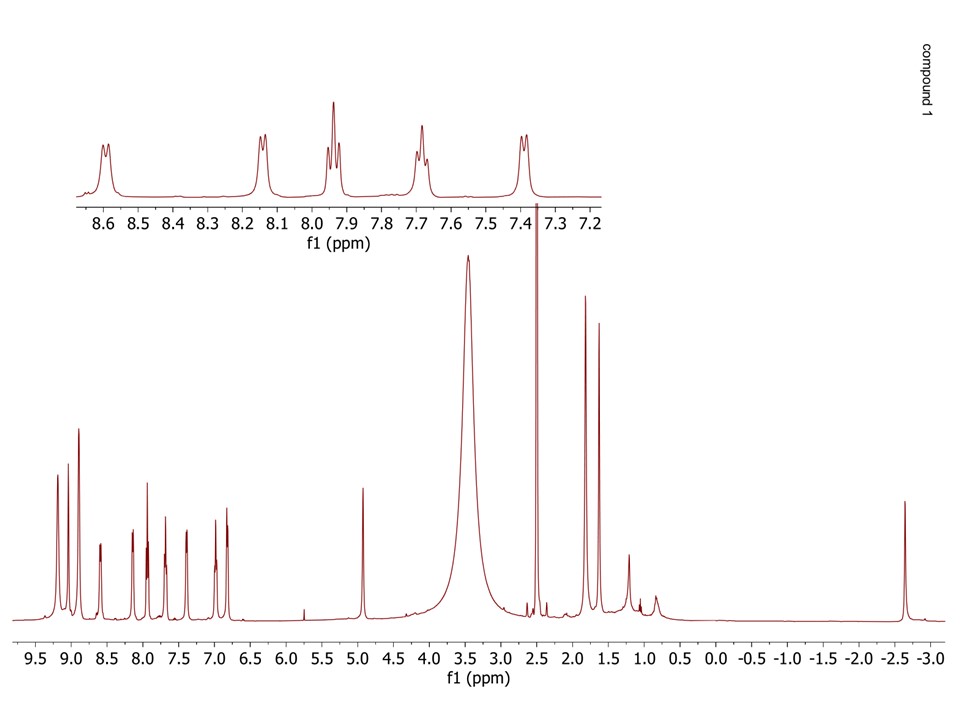


**Figure S50.** ^1^H NMR spectrum (500 MHz, DMSO-*d*6, 300 K) of porphyrin **1**.


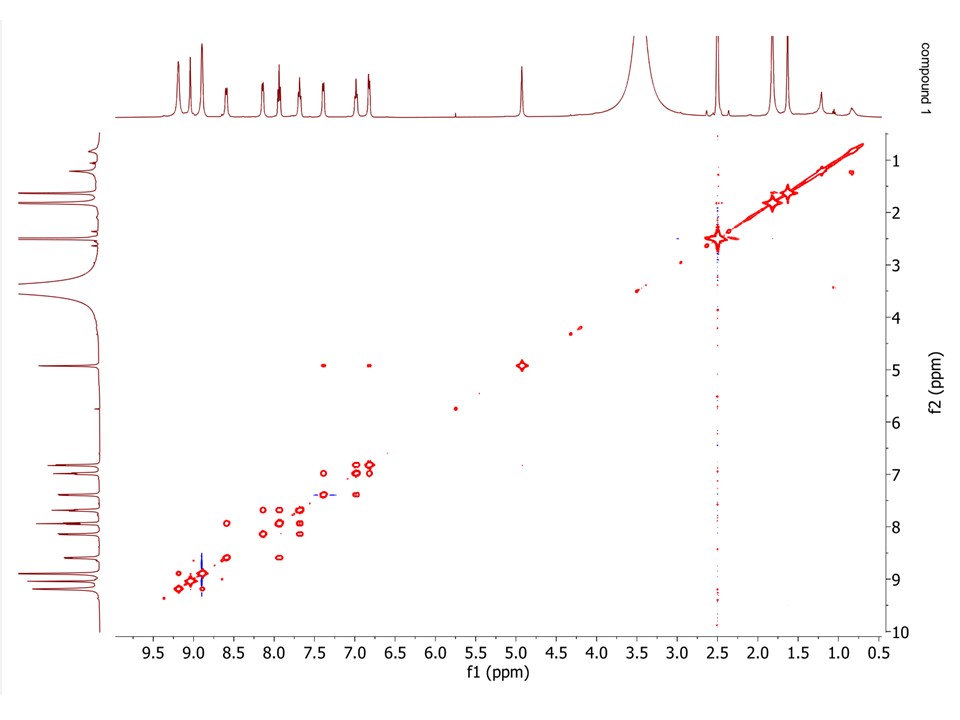


**Figure S51.** 2D COSY ^1^H NMR spectrum (500 MHz, DMSO-*d*6, 300 K) of porphyrin **1**.


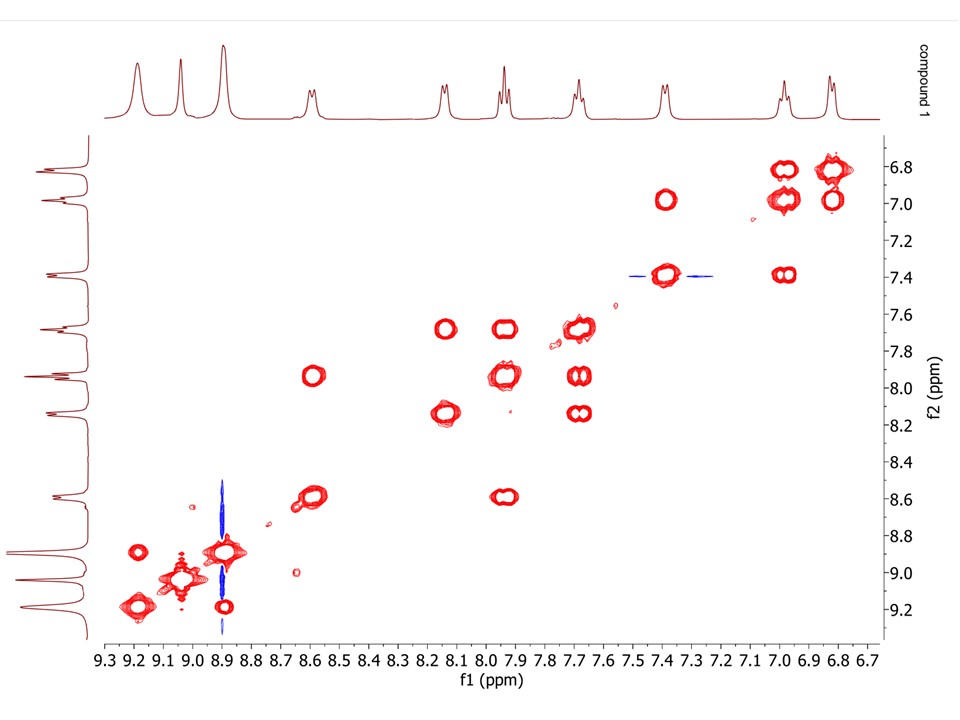


**Figure S52.** 2D COSY ^1^H NMR spectrum (aromatic domain, 500 MHz, DMSO-*d*6, 300 K) of porphyrin **1**.


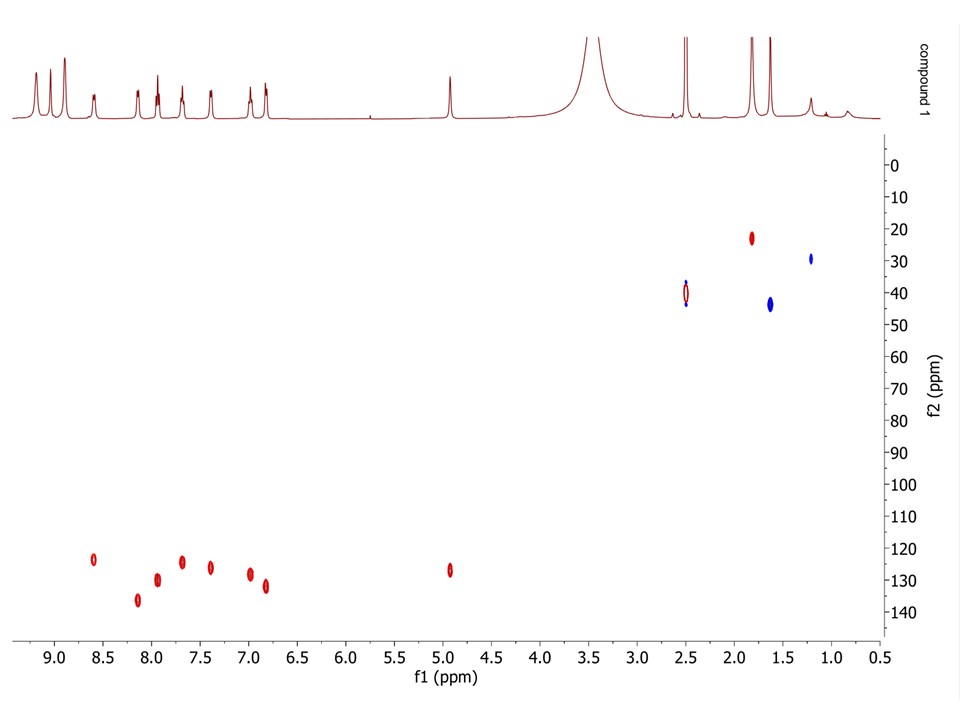


**Figure S53.** 2D HSQC ^1^H NMR spectrum (500 MHz, DMSO-*d*6, 300 K) of porphyrin **1**.


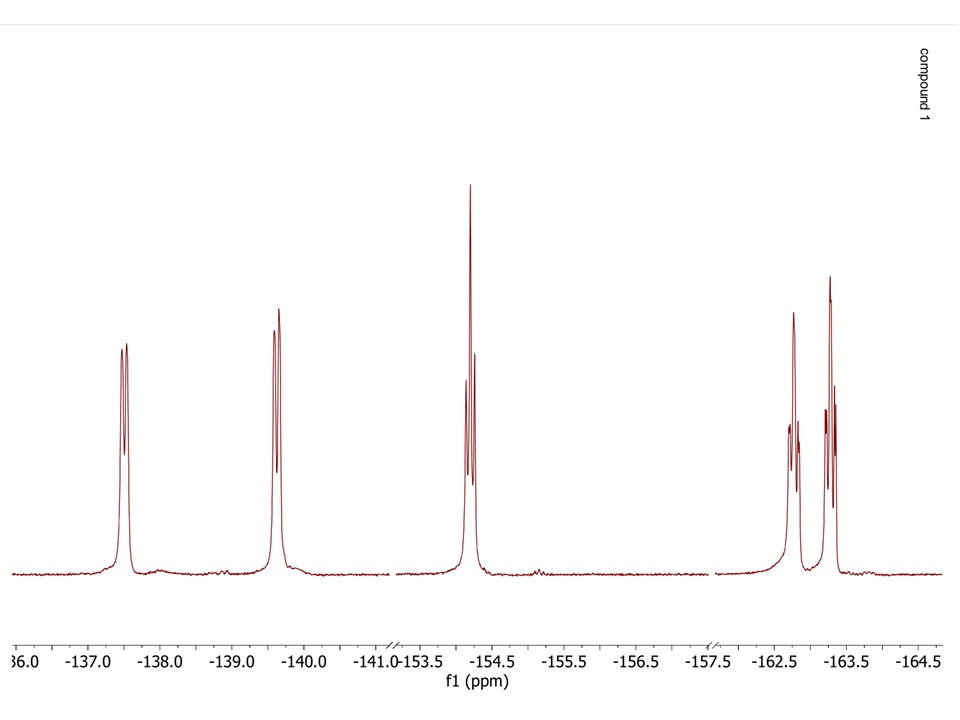


**Figure S54.** ^19^F NMR spectrum (376 MHz, DMSO-*d*6, 300 K) of porphyrin **1**.


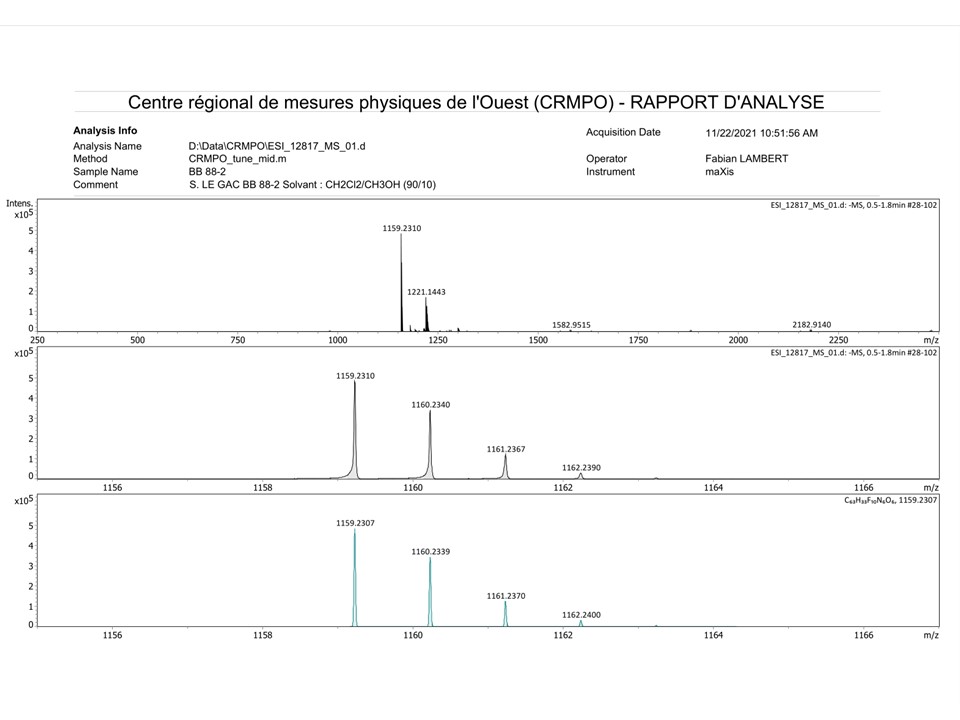


**Figure S55.** ESI-HRMS spectrum of **1.**

**
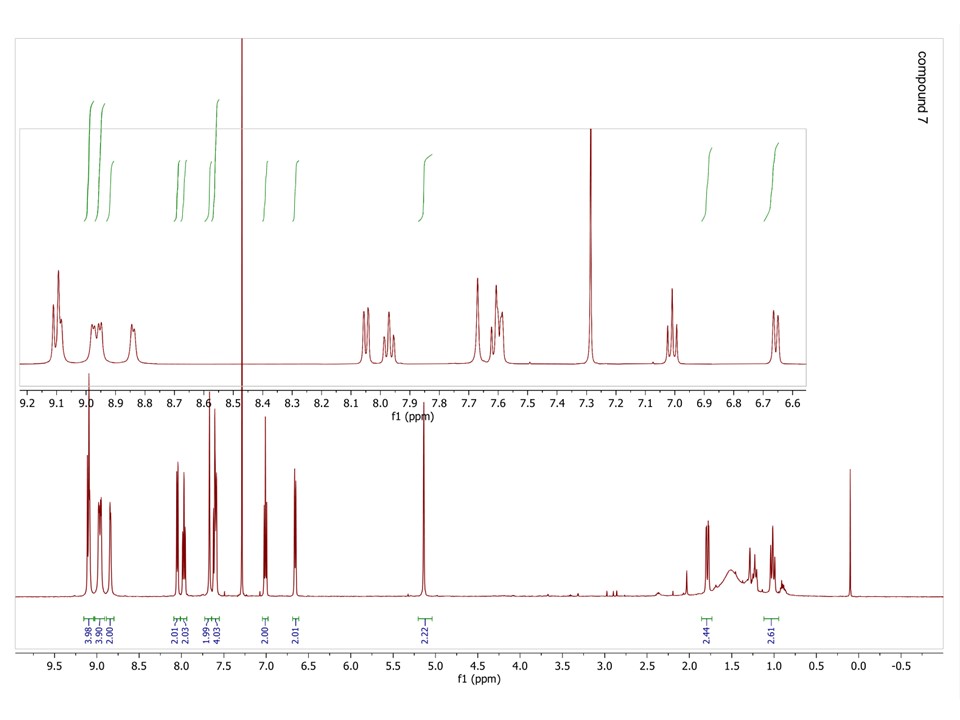
**

**Figure S56.** ^1^H NMR spectrum (500 MHz, CDCl_3_, 300 K) of porphyrin **7**.


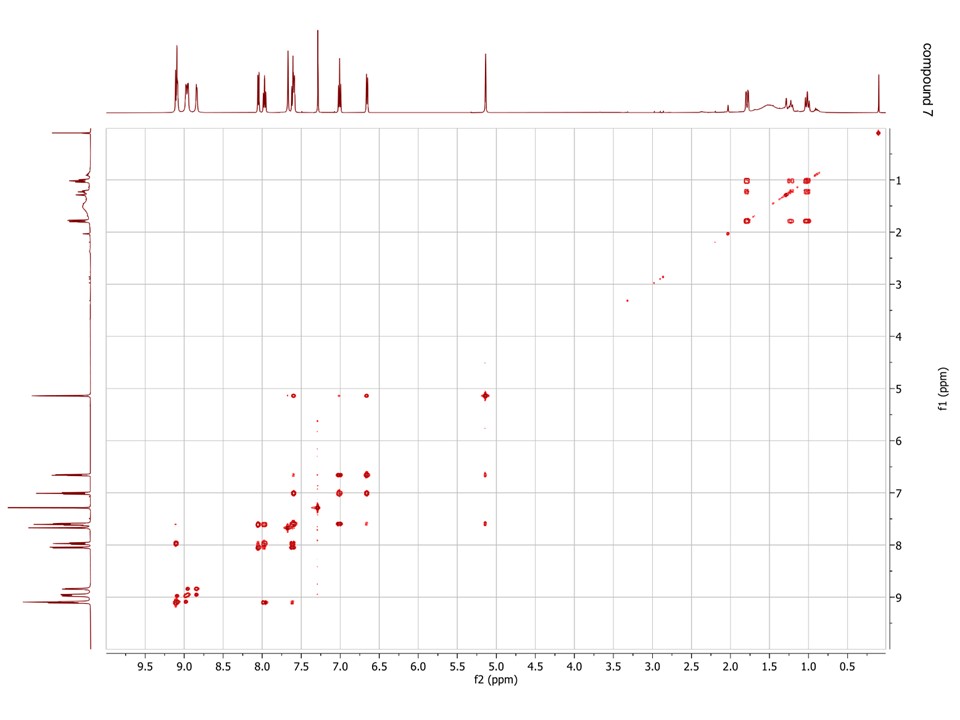


**Figure S57.** 2D COSY ^1^H NMR spectrum (500 MHz, CDCl_3_, 300 K) of porphyrin **7**.


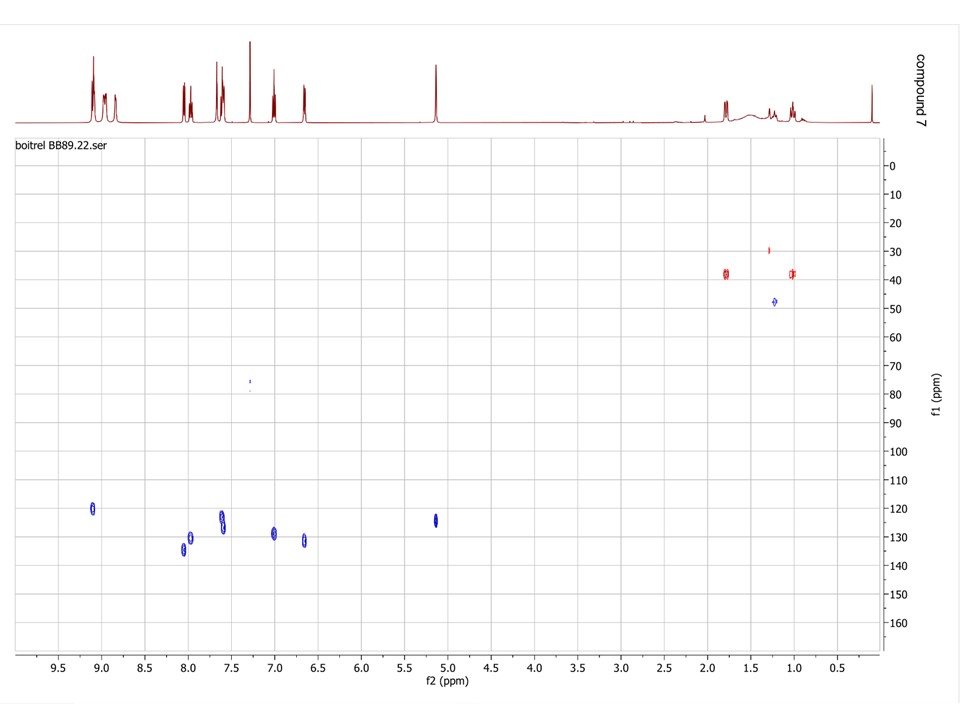


**Figure S58.** 2D HSQC ^1^H NMR spectrum (500 MHz, CDCl_3_, 300 K) of porphyrin **7**.


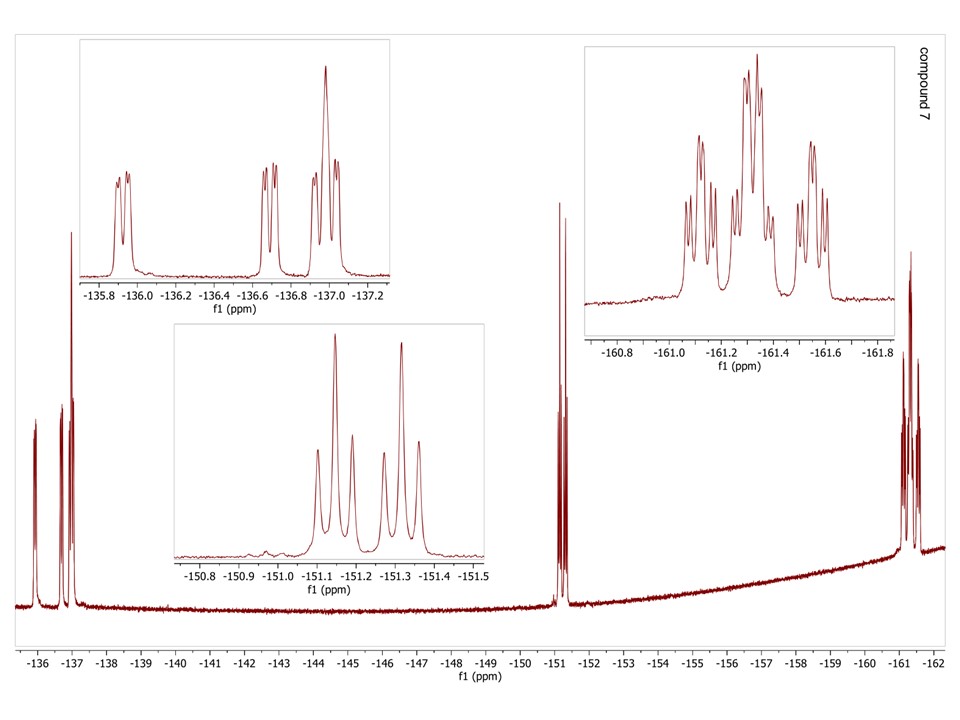


**Figure S59.** ^19^F NMR spectrum (376 MHz, CDCl_3_, 300 K) of porphyrin **7**.


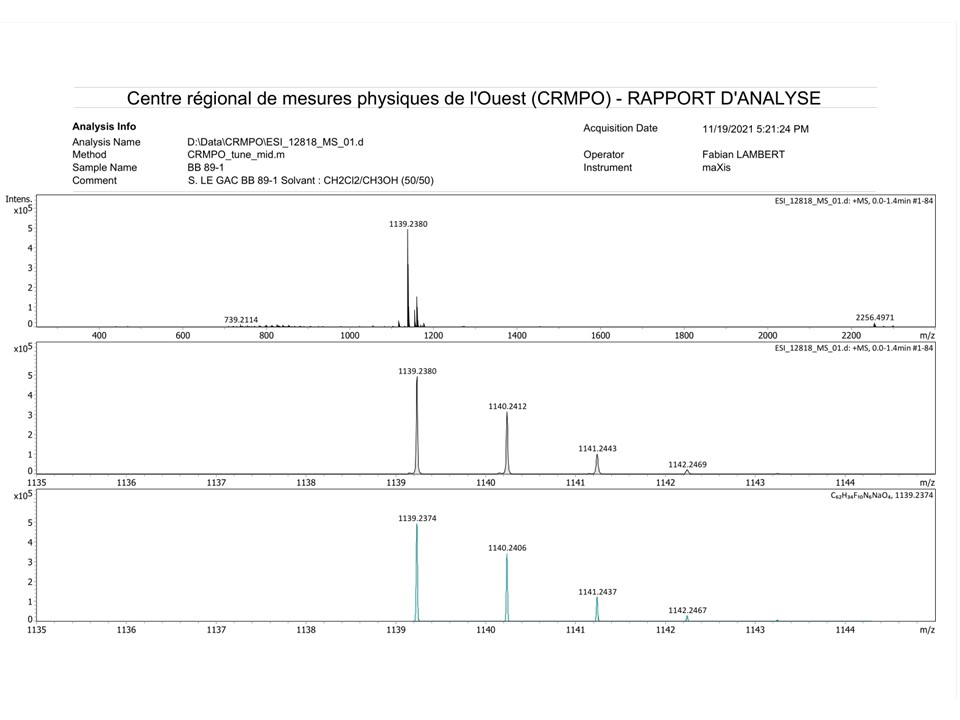


**Figure S60.** ESI-HRMS spectrum of **7**.


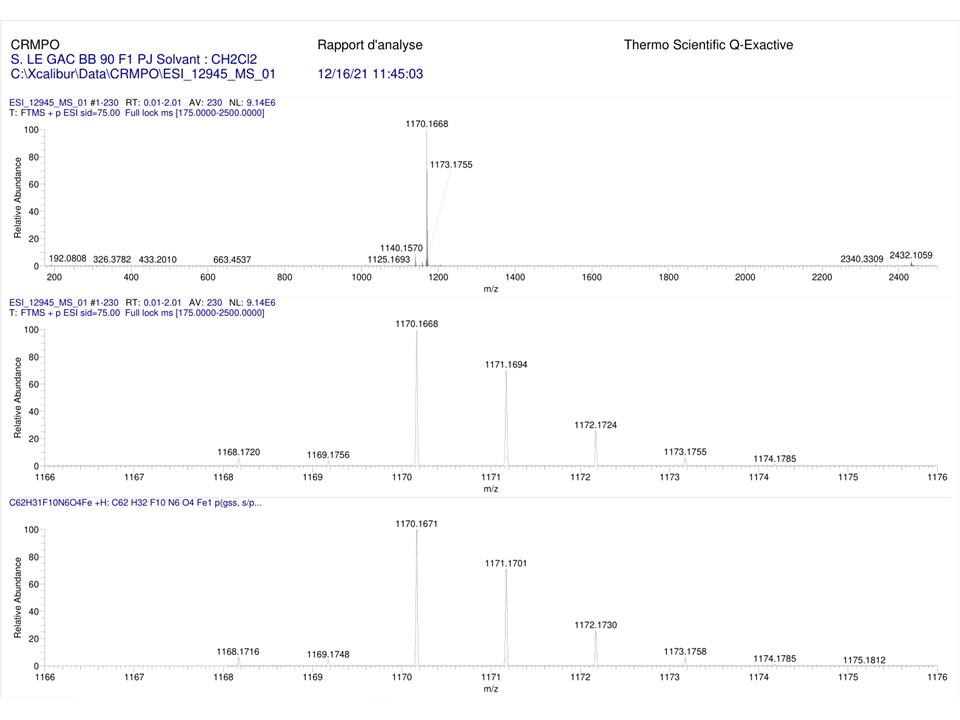


**Figure S61.** ESI-HRMS spectrum of **sc-Fe**.

# References

1. T. Tanaka, A. Osuka, *Chem. Rev.* **2017**, *117*, 2584-2640 [↑](#endnote-ref-1)
2. G. M. Sheldrick, SHELXS-97, Program for Crystal Structure Solution, University of Göttingen, Göttingen, Germany, **1997**. [↑](#endnote-ref-2)
3. G. M. Sheldrick, *Acta Crystallogr., Sect. A: Found. Crystallogr.,* **2008**, *64*, 112-122. [↑](#endnote-ref-3)
4. L. J. Farrugia, *J. Appl. Cryst*. **1999**, *32*, 837. [↑](#endnote-ref-4)
5. C. Costentin, S. Drouet, M. Robert, J.-M. Savéant, *J. Am. Chem. Soc.* **2012**, *134 (27)*, 11235–11242. [↑](#endnote-ref-5)
6. I. Azcarate, C. Costentin, M. Robert, J.-M. Savéant, *J. Phys. Chem. C* **2016**, *120*, 28951–28960. [↑](#endnote-ref-6)
7. I. Azcarate, C. Costentin, M. Robert, J.-M. Savéant, J.-M. *J. Am. Chem. Soc.* **2016**, *138 (51)*, 16639–16644. [↑](#endnote-ref-7)
8. If another material is used for the electrolysis, the diffusion layer of the new surface might be different than it was on the glassy carbon surface used for CVs. Indeed, if the roughness of the electrode is very different, the diffusion of molecules of catalyst around the electrode surface can be different, which can falsify the value of TOF calculated. For example, if have a very rough surface, we can imagine it can hinder the catalysts diffusion by modifying its immediate environment (making the diffusion coefficient different than the D_cat_ of the catalyst free in the solvent). Therefore, we chose to use glassy carbon for both CV and CPE. [↑](#footnote-ref-1)
9. Here, the area A correspond to the area of the working electrode for CPE (different from the one used for the diffusion coefficient calculation). We can note that this TON is counting only the catalyst present in the reaction layer (next to the electrode), and not all the catalyst present in the bulk, because the other catalyst molecules are not participating to the reaction. However, this TON does not accurately take into account the catalysts degradation, but only reflects a “peak TON” that could be achieved for short-term electrolysis if the catalyst is undergoing no degradation (because it is only considering the speed of the reaction itself, at a given potential). In a homogeneous electrolysis setup, degraded inactive catalyst molecules are replaced on the electrode surface by diffusion of catalyst molecules present in the bulk. In case of degradation, inactive molecules will remain in the bulk and this phenomenon will decrease the catalysis intensity after a long-term electrolysis, and the experimental TON should be determined after all catalyst molecules have been degraded. In the case of very stable catalysts (like iron porphyrins), the electrolyte needs to be refreshed multiple times with more substrate (CO_2_, protons), and the electrolysis has to be run for days or weeks before reaching a full degradation, making this method of TON calculation more than tedious. Therefore, comparing TONs can be confusing, depending on how the TON has been calculated (either using the formula shown above, either by simply dividing the quantity of product by the whole quantity of catalyst in the bulk). [↑](#footnote-ref-2)
10. M. J. Frisch, G. W. Trucks, H. B. Schlegel, G. E. Scuseria, M. A. Robb, J. R. Cheeseman, G. Scalmani, V. Barone, B. Mennucci, G. A. Petersson, *et al.* Gaussian, Inc., Wallingford CT, 2016. Gaussian 16, Revision B.01. [↑](#endnote-ref-8)
11. S. Grimme, S. Ehrlich, L. Goerigk, *J. Comput. Chem.* **2011**, *32*, 1456–1465. [↑](#endnote-ref-9)
12. A. D. Becke, *J. Chem. Phys.*, **1993**, *98*, 5648-52. [↑](#endnote-ref-10)
13. F. Weigend and R. Ahlrichs *Phys. Chem. Chem. Phys.*, **2005**, *7*, 3297-305. [↑](#endnote-ref-11)
14. A. V. Marenich, C. J. Cramer, and D. G. Truhlar *J. Phys. Chem. B*, **2009**, ***113*,** 6378-96. [↑](#endnote-ref-12)
15. E. R. Johnson, S. Keinan, P. Mori-Sanchez, J. Contreras-Garcia, A. J. Cohen, W. Yang, *J. Am. Chem. Soc.* **2010**, *132*, 6498-6506 [↑](#endnote-ref-13)
16. W. Humphrey, A. Dalke and K. Schulten *J. Molec. Graphics*, **1996**, *14* 33-38. [↑](#endnote-ref-14)
17. A. A. Isse, A. Gennaro, *J Phys Chem B* **2010**, *114*, 7894-7899. [↑](#endnote-ref-15)
18. J. Song, E. L. Klein, F. Neese, S. Ye, *Inorg. Chem.* **2014**, *53*, 7500-7507. [↑](#endnote-ref-16)
19. P. G. Brewer, *Ann. N. Y. Acad. Sci.* **1978**, *341*, 102-115. [↑](#endnote-ref-17)
